# Thioredoxin-interacting protein (TXNIP) inhibition promotes retinal ganglion cell survival and facilitates M1-like microglial transformation via the PI3K/Akt pathway in glaucoma

**DOI:** 10.1186/s10020-024-01058-5

**Published:** 2024-12-30

**Authors:** Junjue Chen, Huimin Zhong, Bingqiao Shen, Huan Yu, Yang Zhang, Ruiqi Han, Ping Huang, Shouyue Huang, Yisheng Zhong

**Affiliations:** 1 Department of Ophthalmology, Ruijin Hospital Affiliated Medical School, Shanghai Jiaotong University, 197 Ruijin er Road, Shanghai, 200025 China; 2 Department of Ophthalmology, Shanghai General Hospital (Shanghai First People’s Hospital), Shanghai Jiao Tong University School of Medicine, Shanghai, China; 3 Shanghai Key Laboratory for Bone and Joint Diseases, Shanghai Institute of Traumatology and Orthopaedics, Ruijin Hospital Affiliated Medical School, Shanghai Jiaotong University, 197 Ruijin er Road, Shanghai, 200025 China; 4 Department of Ophthalmology, Zhoushan Branch of Ruijin Hospital Affiliated Medical School, Shanghai Jiaotong University, Zhoushan, China

**Keywords:** TXNIP, Microglia, Experimental glaucoma, PI3K/AKT, Neuroinflammation, Energy metabolism

## Abstract

**Background:**

Glaucoma is a group of heterogeneous neurodegenerative diseases with abnormal energy metabolism and imbalanced neuroinflammation in the retina. Thioredoxin-interacting protein (TXNIP) is involved in glucose and lipid metabolism, and associated with oxidative stress and inflammation, however, not known whether to be involved in glaucoma neuropathy and its underlying mechanisms.

**Methods:**

To establish the chronic ocular hypertension (COH) mice model. Western blot, RT-PCR, immunofluorescence and F-VEP were used to detect neuroinflammation level, glial activation and RGCs survival in retina of wild type, TXNIP knockout and MCC950 treatment COH mice. Microglia high-pressure cultured model was constructed. Western blot, RT-PCR and immunofluorescence were used to investigate the proinflammatory cytokines secretion, glucose uptake and phenotype transformation in wild type, TXNIP knockout and overexpressed microglia combined with IL-17A treatment. Finally, we explored the possible underlying mechanisms using relevant pathway inhibitor interventions.

**Results:**

In this study, for the first time we reported that TXNIP expression was remarkably increased in experimental glaucomatous retina of chronic ocular hypertension (COH) mice, and it was mainly expressed in the ganglion cells layer (GCL). In addition, we found that ablation of TXNIP promoted retinal ganglion cells (RGCs) survival and alleviated visual function impairment in experimental glaucoma. Then, we explored the spatiotemporal consistency between glial activation and retinal inflammation levels in COH mice respectively with TXNIP-deficiency and under treatment of a thermo-containing protein domain 3 (NLRP3) inhibitor MCC950, and the results indicated that TXNIP probably mediated neuroinflammation in glaucomatous retina by activating microglia. Furthermore, upregulation of TXNIP was found in pressure-stimulated microglia, whereas silencing TXNIP facilitated microglial polarization trending towards M1 type and reduced glucose transporter-1 (Glut-1) expression on microglia under high pressure in vitro. Moreover, IL-17A was found to play a role in acting synergistically with TXNIP upon the regulation of microglia polarity transformation. Finally, knockout of TXNIP was revealed to promote PI3K phosphorylation, whereas inhibition of PI3K by LY294002 effectively suppressed Glut-1 expression, glucose uptake, and M1-like transformation tendency in microglia obtained from TXNIP-deficiency mice under high pressure stimulation.

**Conclusions:**

TXNIP is significantly involved in the inflammation-related neuropathy of experimental glaucoma and probably facilitates M1-like microglial transformation via PI3K/Akt pathway.

**Supplementary Information:**

The online version contains supplementary material available at 10.1186/s10020-024-01058-5.

## Introduction

Glaucoma is a group of heterogeneous progressive neurodegenerative diseases that is characterized by continued retinal ganglion cell (RGC) degeneration (Quigley and Broman 2006). The internal mechanism of RGC degeneration is incompletely known and is thought to be driven by multiple factors, including metabolic, vascular, oxidative, and inflammatory components (Tezel 2022). It is widely believed that interactions between multiple molecules and cell types contribute to the complicated process of glaucomatous neurodegeneration. Emerging evidence indicates that the reaction of microglia in the retina is an important factor in determining the fate of neurons in glaucoma (Agarwal and Agarwal 2017). Microglia are highly tissue-specific macrophages that play a role in immune surveillance in the retina and central nervous system (CNS) parenchyma (O'Koren et al. 2019). Despite the resilience that allows microglia to maintain homeostasis in the surrounding environment during initial stressful situations, their predominant function may drift in a detrimental direction, exacerbating neurodegeneration in chronic glaucomatous injury (Baudouin et al. 2021). Likewise, microglia-driven neuroinflammation is spatially consistent with glaucomatous RGC degeneration. In human glaucoma, degenerative changes in the optic nerve head are associated with amoeboid microglia, which are immunopositive for the inflammatory mediators TNF-α, COX-1 and NOS-2 (Yuan and Neufeld 2001). Moreover, in regard to microglia-mediated neuroinflammation, it is necessary to mention two subtypes of microglia, namely, the M1 and M2 phenotypes. M1 microglia are classically activated by LPS and IFNγ to produce proinflammatory factors and consequently increase neurotoxicity, while M2 microglia are alternatively activated by IL-4 to facilitate anti-inflammatory effects and promote tissue remodeling (Agarwal and Agarwal 2017). In the early stage of experimental glaucoma, the proportion of CD206^+^ M2-like microglia is briefly elevated, and the percentage of CD86^+^ M1-like microglia remains at relatively high levels the whole time (Hu et al. 2021). However, the specific mechanisms underlying the phenotypic transformation of microglia in experimental glaucoma have not been elucidated, and further research is needed.

Thioredoxin-interacting protein (TXNIP) is a member of the α-arrestin family of proteins and is highly expressed in the retina, including RGCs, astrocytes, retinal pigment epithelium (RPE) cells and microglia (Tsubaki et al. 2020; Simó et al. 2010). This molecule is a critical regulator of mitochondrial oxidative phosphorylation and fatty acid β oxidation, which is essential for proper mitochondrial function (Tsubaki et al. 2020). Since healthy mitochondria are critical for the regulation of microglial polarization under inflammatory conditions, dysfunctional mitochondria stimulate and aggravate inflammatory responses from microglia in neurodegenerative diseases (Tezel 2020; Wong et al. 2020). Moreover, TXNIP is directly involved in the inflammatory reaction by combining with Nucleotide binding domain, leucine-rich family, thermo-containing protein domain 3 (NLRP3) inflammasome complexes. Although there is evidence suggesting that TXNIP is involved in retinal diseases, little is known about its role in experimental glaucoma (Singh 2013; Devi et al. 2019).

In our previous study, we found that IL-17A was dynamically involved in regulating the transformation of the microglial phenotype in experimental glaucoma, but the underlying mechanism was not fully elucidated (Chen et al. 2023). Given the importance of TXNIP’s role in the regulation of immune inflammation and the maintenance of mitochondrial function, we suspected that TXNIP and IL-17A synergistically participate in the modulation of the microglial phenotype under pathologically high intraocular pressure (IOP), as in the context of glaucoma. In this study, we induced experimental glaucoma in forms of a mouse model of chronic ocular hypertension (COH) and a pressurized cell culture system for separate observation of retinal microglia to explore the specific role of TXNIP in the relationship with microglial activities. Our results will help to improve the understanding of the pathogenesis of glaucoma and potentially inspire the development of therapeutic strategies focused on glaucoma optic neuropathy as well as other neurodegenerative disorders.

## Materials and methods

### Animals

Male wild-type (WT) C57BL/6 J mice (6–8 weeks old, 19–25 g weight) and TXNIP knockout (KO) mice (6–8 weeks old, 19–25 g weight) on a C57BL/6 J background were obtained from Charles River Laboratories (Zhejiang, China, licence number: SCXK (Zhe)2019–0001). All animal experimental protocols were approved by the Animal Care and Use Committee of Ruijin Hospital affiliated to Shanghai Jiao Tong University School of Medicine and adhered to the Association for Research in Vision and Ophthalmology Statement on the Use of Animals in Ophthalmic and Vision Research. Mice were fed ad libitum, and their environment was maintained at approximately 21 °C on an alternating cycle of 12 h of light and 12 h of darkness. A total of 600 mice were used in this study.

### Surgical induction of chronic ocular hypertension in mice

COH was induced in the right eyes of mice based on the protocol described in our previous study (Chen et al. 2021). Briefly, the mice were anesthetized by intraperitoneal administration of 80 mg/kg ketamine hydrochloride and 16 mg/kg xylazine (Sigma-Aldrich, St. Louis, MO, USA). A drop of 0.5% proparacaine hydrochloride (Bausch & Lomb, Tampa, FL, USA) was used for topical anesthesia of the ocular surface. A HyStem Cell Culture Scaffold Kit (HCCS; Sigma-Aldrich) was premixed and then immediately injected (3 µL) into the anterior chamber using a 31G needle (Hamilton Bonaduz AG, Switzerland) in the operation group. The same operative procedure was performed in the control group (Sham group), except that an equivalent volume of phosphate-buffered saline (PBS) instead of crosslinking hydrogel was injected into the anterior chamber of the right eye of each mouse.

### The measurement of intraocular pressure (IOP)

The mice were given rapid general anesthesia by isoflurane (2%−4%, Sigma-Aldrich) inhalation, and the IOP was measured using a hand-held rebound tonometer (I-care Tonolab, Helsinki, Finland). The time of the IOP measurement was set between 10 a.m. and 2 p.m. IOP was measured immediately before the surgery, one day postoperatively and each week postoperatively until the end of the planned experimental period (at the fourth week after COH induction).

### Animal grouping and drug administration

The mice were divided into groups according to a randomization procedure (http://www.randomizer.org/): the control, COH (WT mice or TXNIP KO mice) + PBS, COH (WT mice) + MCC950 (Selleck Chemicals, Houston, TX, USA). Intravitreal injections of 1 µL of MCC950 (10 mM) were performed three days prior to COH induction and weekly after surgery until the end point of the experimental sampling period (at the fourth week after COH induction). The first injection timepoint was three days before surgery, and the injections were repeated once weekly after surgery. Normal saline was used as a vehicle control. Intravitreal injections were performed using a 32G needle (Hamilton Bonaduz AG).

### Primary retinal microglial cell cultures

Primary retinal microglial cell cultures were obtained from WT or TXNIP KO C57BL/6 J mice. Briefly, a cell suspension of dissociated retinas from one-day-old mice was plated in T75 flasks (with the cells from 24 retinas in each flask) and maintained in DMEM/F-12 medium (Gibco, Invitrogen, Carlsbad, CA, USA) supplemented with 10% fetal bovine serum (FBS; EpiZyme, Shanghai, China) at 37 °C in a humidified incubator with 5% CO_2_. The cultures were shaken for seven hours at 110 rpm and 37 °C after 14 days. The microglia were collected from the cell supernatant, and then the collected microglia were resuspended and plated at a density of 3 × 10^6^ cells/well in 10-cm^2^ cell culture dishes and cultured at 37 °C in a humidified atmosphere of 5% CO_2_. The purity of the isolated microglia was assessed using immunocytochemistry with an anti-IBA-1 antibody.

### Cultures grouping and treatment

Isolated retinal microglia cultures were divided randomly into different groups as follows and compared within separate subsets respectively as designed for different targets of observation: microglia (WT) + PBS, microglia (WT) + recombinant mouse IL-17A (rmIL-17A; 100 ng/mL; R&D, Emeryville, CA, USA), microglia (TXNIP KO) + rmIL-17A (100 ng/mL) cultured under normal pressure at 37 °C in a humidified atmosphere of 5% CO_2_; microglia (WT) + PBS, microglia (WT) + rmIL-17A (100 ng/mL), microglia (WT) + IL-17A neutralizing antibody (IL-17A Nab; 50 ng/mL; R&D), microglia (TXNIP KO) + rmIL-17A (100 ng/mL), microglia (TXNIP KO) + LY294002 (25μM; Beyotime, Shanghai, China),cultured under high pressure at 37 °C in a pressurized cell culture system (Flexcell FX-5000, Flexcell International Corporation, Burlington, NC, USA).

### Pressurized cell culture

Isolated retinal microglia were resuspended in mixed pressurized culture medium based on the protocol described in our previous study (Chen et al. 2023). Briefly, the mixed cell suspension was added to a BioPress compression culture plate (Flexcell International Corporation) at a density of 250 μL/well and cultured at 37 °C in a humidified atmosphere of 5% CO_2_ overnight. Pressurized cell culture was performed using a Flexcell FX-5000 compression system. The pressure of the compression system was set to 37.5 mmHg (5 kPa) at a frequency of 0.1 Hz, and the maximum duration of high-pressure culture was set to 8 h.

### Western blotting analysis

Retinas or cells were lysed in radioimmunoprecipitation assay buffer (RIPA; Sigma-Aldrich) supplemented with a protease inhibitor cocktail (Sigma-Aldrich) at a 100:1 ratio. The samples were centrifuged, and the protein concentrations in the supernatant were quantified. SDS polyacrylamide gel electrophoresis was used to separate the proteins of different molecular weights in samples (containing equal amounts of protein), and the target proteins were then electrotransferred to polyvinylidene fluoride membranes. The membranes were blocked with rapid blocking solution at room temperature for 10 min and incubated with the corresponding primary antibodies of rabbit anti-actin (ab179467,1:5000; Abcam, Cambridge, UK), rabbit anti-TNF-α (ab215188, 1:1000; Abcam), rabbit anti-IL-1β (ab234437, 1:1000; Abcam), rabbit anti-IBA-1 (ab178846, 1:1000; Abcam), rabbit anti-IL-17A (ab79056, 1:1000; Abcam), rabbit anti-CD68 (ab303565, 1:1000; Abcam), rabbit anti-CD206 (ab64693, 1:1000; Abcam), rabbit anti-inducible nitric oxide synthase (iNOS; ab178945, 1:1000; Abcam), rabbit anti-arginase 1 (ARG1; ab233548, 1:1000; Abcam), rabbit anti-CD86 (ab112490, 1:1000; Abcam), rabbit anti-glial fibrillary acidic protein (ab7260, GFAP; 1:1000; Abcam), rabbit anti-glutamine synthetase (ab73593, GS; 1:1000; Abcam), rabbit anti-Brn3a (ab245230, 1:1000; Abcam), rabbit anti-TXNIP (14715S, 1:1000; Cell Signaling Technology, Boston, MA, USA), rabbit anti-p38(8690, 1:1000; Cell Signaling Technology), rabbit anti-p-p38 (4511, 1:1000; Cell Signaling Technology), rabbit anti-extracellular signal-regulated kinase 1/2 (ERK1/2; 8544, 1:1000; Cell Signaling Technology), rabbit anti-p-ERK1/2 (4370, 1:1000; Cell Signaling Technology), rabbit anti-Akt ( AA326, 1:1000; Beyotime), rabbit anti-p-Akt-ser473 (AA329, 1:1000; Beyotime), rabbit anti-p-Akt-thr308 (AA331, 1:1000; Beyotime), rabbit anti-PI3 Kinase p85 (4257 T, 1:1000; Cell Signaling Technology), rabbit anti-p-PI3 Kinase p85/p55 (4228 T, 1:1000; Cell Signaling Technology), rabbit anti-AMPKα (5831S, 1:1000; Cell Signaling Technology) or rabbit anti-p-AMPKα(50081S, 1:1000; Cell Signaling Technology) at 4 °C overnight. Then, the membranes were incubated with appropriate horseradish peroxidase-conjugated goat anti-rabbit (ab97051, 1:1000; Abcam) secondary antibodies at room temperature for 1 h. Protein bands on the membranes were visualized with an ImageQuant LAS 4000 Mini system (GE Healthcare Bio Sciences, Piscataway, NJ, USA) and quantitatively analyzed with the application of ImageJ software (version 2.1.0; Media Cybernetics, Silver Springs, MD, USA).

### Real-time RT-PCR analysis

Total RNA was extracted from samples using TRIzol reagent (Invitrogen, Waltham, MA, USA) according to the manufacturer’s protocol. A NanoDrop (Thermo Fisher Scientific, Waltham, MA, USA) instrument was used to determine the concentration and purity of the RNA. RNA was reverse transcribed into cDNA using a PrimeScript RT Master Mix kit (Takara, Tokyo, Japan). Quantitative RT-PCR was performed on each RNA sample in a volume of 10 μL using a TB Green Premix Ex Taq kit (Takara) on a 7500 Real-Time PCR system (Applied Biosystems, Waltham, MA, USA). Relative gene expression was quantified using the 2^−ΔΔCt^ method, and β-actin was used as an internal reference gene. Six samples were used for each analysis, and the experiment was repeated three times. The primers designed for use in this study were as follows:

β-actin: forward, 5′-GCAGATGTGGATCAG CAAGC-3′ and reverse, 5′-GCAGCTCAGTAACAG TCCGC′;

IL-17A: forward, 5′-CACCGCAATGAA GACCCTGA-3′ and reverse, 5′-TTCCCTCCGCATTGA CACAG-3′;

IL-1β: forward, 5′-TGCCACCTTTT GACAGTGATG-3′ and reverse, 5′-AAGGTCCACGGGAAAGACAC-3′;

Arg-1: forward, 5′-CATATCTGCCAAAGACATCGTG-3′ and reverse, 5′-GACATCAAAGCTCAGGTGAATC-3′;

iNOS: forward, 5′- ACTCAGCCAAGCCCTCACCTAC-3′ and reverse, 5′-TCCAATCTCTGCCTATCCGTCTCG-3′;

CD206: forward, 5′-CCTATGAAAATTGGGCTTACGG.

−3′ and reverse, 5′-CTGACAAATCCAGTTGTTGAGG-3′;

CD68: forward, 5′-GAAATGTCACAGTTCACACCAG-3′ and reverse, 5′- GGATCTTGGACTAGTAGCAGTG-3′;

CD86: forward, 5′- ACGGAGTCAATGAAGATTTCCT-3′ and reverse, 5′- GATTCGGCTTCTTGTGACATAC-3′;

IBA-1: forward, 5′- ATTATGTCCTTGAAGCGAATGC-3′ and reverse, 5′- TCTCAAGATGGCAGATCTCTTG-3′; and.

TNF-α: forward, 5′- ATGTCTCAGCCTCTTCTCATTC-3′ and reverse, 5′- GCTTGTCACTCGAATTTTGAGA-3′.

TXNIP: forward, 5′-GACGATGTGGACGACTCTCAAGAC-3′and reverse, 5′-GTTGTTGTT AAGGACGCACGGATC-3′;

GFAP: forward, 5′-GAGAACAACCTGGCTGCGTATAGAC-3′and reverse, 5′- CCTCCTCCAGCGATTCAACCTTTC-3′;

GS: forward, 5′-TCCACGAAACCTCCAACATCAACG-3′and reverse, 5′- GTCTTCAAAGTAGCCCTTCTTCTCCTG −3′;

Brn3a: forward, 5′-ACGCTCTCGCACAACAACATGATC-3′and reverse, 5′- GCTCCGGCTTGTTCATTTTCTCAC-3′;

### Flash visual-evoked potential examination

Flash visual-evoked potential (F-VEP) examination was performed according to the protocol described in our previous study (Chen et al. 2023). Briefly, the recording and reference electrodes were placed under the scalp at the occipital tuberosity and under the skin of the nose, respectively, in the anesthetized mice. The ground electrode was placed at the mastoid process, and then the eyes were adapted to the dark for 15 min. The contralateral eye was covered by an opaque black blindfold, and F-VEP (UTAS-E3000LKC, Multifocal Visual Diagnostic Test System, LKC Technologies, Gaithersburg, MD, USA) detection of the tested eye was started with flashing light stimuli at an intensity of 3.12 cd s^−1^ m^−2^. The high and low frequencies were 300 Hz and 0.1 Hz, respectively. The F-VEP inspections were carried out before the operation and again 4 weeks postoperatively, with at least three consecutive measurements being performed each time.

### Immunofluorescence analysis of retinal whole mounts

The right eyes were enucleated from mice and further immersed in 4% PFA at 4 °C for 1 h. The eyecups were prepared, and the whole retinas were isolated and permeated with cold 0.25% Triton X-100 (Sigma-Aldrich) for 30 min and blocked in 5% BSA (Sigma-Aldrich) at room temperature for 1 h. The retinal whole mounts were incubated with the primary antibody rabbit anti-IBA-1 (ab178846, 1:500; Abcam), or rabbit anti-Brn3a (ab245230, 1:100; Abcam) at 4 ℃ overnight, followed by incubation for 1 h with fluorescence-conjugated secondary antibody (A31572, 1:500; Thermo Fisher Scientific) at room temperature. The retinas were cut into the shape of a four-leaf clover and mounted with fluorescence mounting medium (Dako, Carpinteria, CA, USA). Fluorescence images were obtained using a confocal laser-scanning microscope (LSM 510 META; Zeiss, Jena, Germany).

### Immunofluorescence analysis of retinal cryosections

The right eyes were enucleated from euthanized mice and immersed in 4% PFA at 4 °C overnight. The eyecups were prepared and graded sucrose solutions were used for tissue dehydration. Then, the eyecups and optic nerves were embedded in an embedding agent (optimum cutting temperature compound, OCT; Sakura Finetek, Torrance, CA, USA) and frozen. A Leica microtome (CM1950, Wetzlar, Germany) was used to make ten-micrometer-thick tissue cryosections. The slices were permeated with cold 0.25% Triton X-100 (Sigma-Aldrich) for 30 min and blocked in 5% BSA (Sigma-Aldrich) at room temperature for 1 h. The retinal slices were respectively incubated with the primary antibodies of rabbit anti-GFAP (ab207165, 1:500; Abcam), mouse anti-TXNIP (ab210826, 1:500; Abcam), rabbit anti-GS (ab73593, 1:500; Abcam), or rabbit anti-IBA-1(ab283346, 1:500; Abcam) combined with mouse anti-CD68 (ab283654, 1:50; Abcam), and the optic nerve slices were incubated with a primary antibody of neurofilament heavy polypeptide (NEFH; ab207176, 1:100; Abcam) at 4 °C overnight, followed by incubation for 1 h with fluorescence-conjugated secondary antibodies corresponding to the species of the primary antibodies (A31572, A11055 or ab150165, 1:500; Thermo Fisher Scientific) at room temperature. After washed thoroughly, the sections were counterstained with 4′,6-diamidino-2-phenylindole (DAPI; Beyotime) for nuclear staining and mounted with fluorescence mounting medium (Dako). The fluorescence images were obtained using a confocal laser-scanning microscope (LSM 510 META; Zeiss).

### Immunofluorescence analysis for primary retinal microglia

Primary retinal microglial cells were plated in 24-well plates with pretreated coverslips at cell density of about 1 × 10^5^/well. The cells were washed using PBS for 3 times and immersed in 4% PFA for 15 min at room temperature. Then the microglia were permeated with cold 0.25% Triton X-100 (Sigma-Aldrich) for 30 min and blocked in 5% BSA (Sigma-Aldrich) at room temperature for 1 h. The retinal microglia were respectively incubated with the primary antibodies of rabbit anti-IBA-1 (ab178846, 1:500; Abcam) combined with mouse anti-iNOS (ab210823, 1:500; Abcam), or rabbit anti-IBA-1 (ab178846, 1:500; Abcam) combined with mouse anti-Arg-1(ab239731, 1:1000; Abcam) at 4 °C overnight, followed by incubation for 1 h with fluorescence-conjugated secondary antibodies corresponding to the species of the primary antibodies (A31572, A11055 or ab150165, 1:500; Thermo Fisher Scientific) at room temperature. After washed thoroughly, the microglia were counterstained with 4′,6-diamidino-2-phenylindole (DAPI) for nuclear staining and the coverslips mounted with fluorescence mounting medium (Dako). The fluorescence images were obtained using a confocal laser-scanning microscope (LSM 510 META; Zeiss).

### Immunofluorescence quantification

For retinal whole mounts, the method for quantification was as follows. The RGCs and microglia located within 1/6, 3/6 and 5/6 of the retinal radius, with the optic papilla as the center point, were counted in each quadrant (as defined by anatomical orientation). The average RGC and microglial counts in the same size of area in different quadrants were calculated. Three images were acquired per retinal radius, for a total of 36 images per retinal whole mount. Images of retinal cryosection slices were obtained with the same exposure time and intensity. For image quantification, three randomly selected nonoverlapping subranges of 0.10 mm^2^ within a cryosection were examined; a total of 12 values for each staining target were obtained from one eye of each mouse, and these values were averaged for the individual mouse. Immunofluorescence quantification was performed according to a previous study (Reboussin et al. 2022). Image analyses were performed using ImageJ software (version 2.1.0; Media Cybernetics). The specific method was as follows: the images were converted into grayscale and the raw integrated density was quantified to obtain the sum of the pixel values.

### RNA isolation and library preparation

Total RNA was extracted using TRIzol reagent (Invitrogen) according to the manufacturer’s protocol. A NanoDrop 2000 spectrophotometer (Thermo Fisher Scientific) was used to measure the purity and quantity of the total RNA. An Agilent 2100 Bioanalyzer (Agilent Technologies, Santa Clara, CA, USA) was used to assess the RNA integrity. Libraries were constructed using the TruSeq Stranded mRNA LT Sample Prep Kit (Illumina, San Diego, CA, USA) according to the manufacturer’s instructions.

### RNA sequencing and differentially expressed genes analysis

The libraries were sequenced on an Illumina HiSeq X Ten platform, and 150 bp paired-end reads were generated. Approximately 7.8 G of raw reads for each sample were generated. Raw data (raw reads) in fastq format were first processed using Trimmomatic, and low-quality reads were removed to obtain clean reads. Approximately 7.2 G of clean reads for each sample were retained for subsequent analyses. The clean reads were mapped to the mouse genome using HISAT2. The fragments per kilobase of exon per million mapped fragments (FPKM) value of each gene was calculated using Cufflinks, and the read counts for each gene were obtained using HTSeq-count. Differential expression analysis was performed using the DESeq (2012) R package. A P value < 0.05 and a fold-change > 2 or < 0.5 were set as the thresholds for significantly differential expression. Hierarchical cluster analysis of differentially expressed genes (DEGs) was performed to show the expression patterns of genes in different groups and samples.

### Gene overexpression with a DNA-lipid complex

Primary retinal microglia were plated in 12-well plates at a cell density of approximately 3 × 10^5^/ well. When cells reached 80% confluence, a DNA-lipid complex targeting TXNIP and a negative control complex (BioeGene, Shanghai, China) were transiently transfected into microglia using Lipofextamine 3000 plasmid transfection reagent (Invitrogen) according to the manufacturer’s protocol.

### Statistical analysis

The results were presented as mean ± standard deviation (SD). Statistical analysis was performed using SPSS software (version 22.0; IBM Corporation, Armonk, NY, USA). The normality of the data distribution was tested using the Shapiro–Wilk test. Comparisons of experimental data were performed using a two-tailed independent-samples t test between two groups, or with one-way ANOVA followed by the Least Significant Difference test to determine differences between means in multiple sets of experiments. P < 0.05 indicated that a difference was statistically significant.

## Results

### Inhibition of TXNIP had a neuroprotective effect in COH

**Fig. 1:**
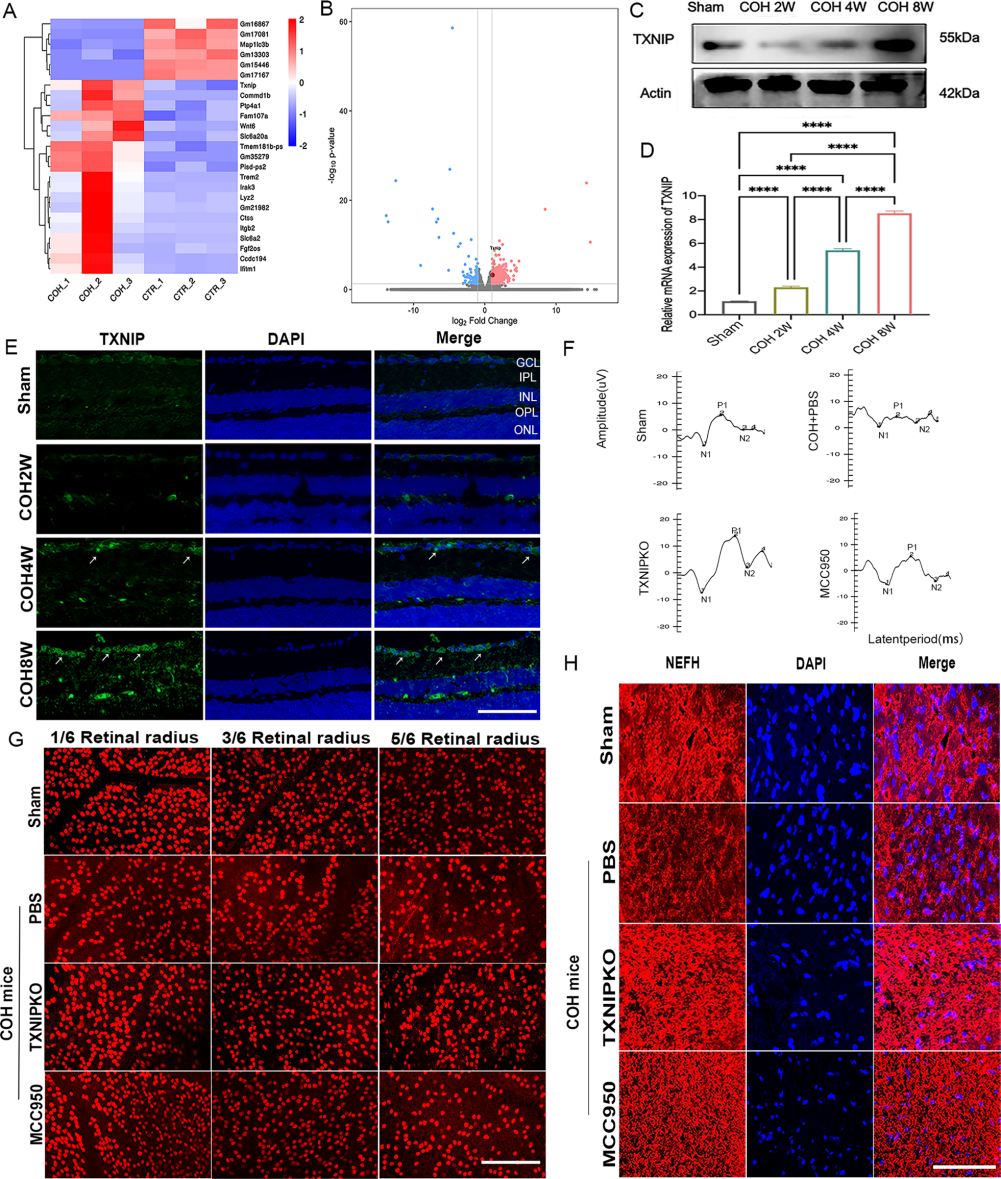
TXNIP was involved in the neuropathy of experimental glaucoma. **A**, **B** Heat map and a volcano plot showed the fold-change in gene expression (log2 scale) and significance (− log10 scale) between the COH groups and control groups. Upregulated genes, red; downregulated genes, green. P values were adjusted based on Bonferroni correction. **C**, **D** Western blotting analysis and real-time RT-PCR analysis of TXNIP at 0, 2, 4, and 8 weeks of COH. **E** Immunofluorescent staining of TXNIP (green) in retinal slices at 0, 2, 4, and 8 weeks of COH (white arrows represent RGCs that expressing TXNIP). **F** F-VEP test at 4 weeks after COH modeling in different groups. **G** Immunofluorescent staining of Brn3a in retinal whole mounts at 4 weeks of COH mice in different groups. H. Immunofluorescent staining of NEFH in retinal whole mounts at 4 weeks of COH mice in different groups.(magnification 200 × , scale bar = 50 μm). n = 8 per group for immunofluorescent staining, real-time RT–PCR, Western blotting. n = 12 per group for the F-VEP test. One-way ANOVA was performed. *p < 0.05, **p < 0.01, ***p < 0·001 and ****p < 0.0001, ns, no significance. Bars represent the mean ± SD. NEFH, neurofilament heavy polypeptide. ONL, outer nuclear layer; OPL, outer plexiform layer; INL, inner nuclear layer; IPL, inner plexiform layer; GCL, ganglion cell layer. MCC950, PBS and TXNIPKO which means WT COH mice intravitreal injection with MCC950 or PBS,or TXNIPKO COH mice, respectively

To first investigate the physiopathological function of TXNIP in experimental glaucoma, we applied transcriptome sequencing using the Illumina HiSeq X Ten platform. Analysis was performed on retinas from normal and COH mice. We observed that TXNIP gene expression was significantly elevated in glaucomatous retinas (FPKM value were 4.35 (COH_1), 6.72 (COH_2), 5.48 (COH_3), 3.25 (CTR_1), 2.53 (CTR_2), 2.03 (CTR_3), respectively; log2 (fc) = 1.08 and p = 0.00, Fig. 1A, B). Next, we verified the RNA-sequencing data using molecular biological methods; the results indicated that the TXNIP protein was mainly expressed in the ganglion cell layer (GCL, Fig. 1E) and that its expression increased gradually with prolonged exposure to pathologically elevated IOP (Fig. 1C, D, Supplementary Fig. 1A).

We further explored the role of TXNIP in glaucomatous neuropathy. Since TXNIP mediates inflammatory responses by binding the NLRP3 inflammasome, we also used intravitreal injection of MCC950, an NLRP3 inhibitor, to indirectly observe the relationship between TXNIP and neuropathy in glaucoma. During the experiment time, there was no statistically significant difference of the mean IOP among the PBS, TXNIPKO, and MCC950 group at the same measurement time point (Supplementary Fig. 1F). The data indicated that both ablation of TXNIP and intravitreal injection of MCC950 markedly promoted the RGC survival in all three of the examined retinal positions in COH mice after four weeks of IOP elevation (Fig. 1G, Supplementary Fig. 1B). To analyze the loss of optic nerve axons in COH mice, immunofluorescence staining for NEFH in optic nerves was performed. The mean raw integrated density of NEFH was significantly increased in both MCC950-treated and TXNIP-deficient COH mice (Fig. 1H, Supplementary Fig. 1E). A similar improvement in visual function was detected with the F-VEP tests, and the results showed that the prolongation of the latent period and the decrease in amplitude were alleviated in both MCC950-treated and TXNIP-deficient COH mice (Fig. 1F, Supplementary Fig. 1C-D). Taken together, these data suggested that either direct knockout of TXNIP or blockade of the NLRP3 pathway, which is essential for the effect of TXNIP, could exert a neuroprotective effect in experimental glaucoma.

### TXNIP mediated neuroinflammation in experimental glaucoma by activating microglia

**Fig. 2:**
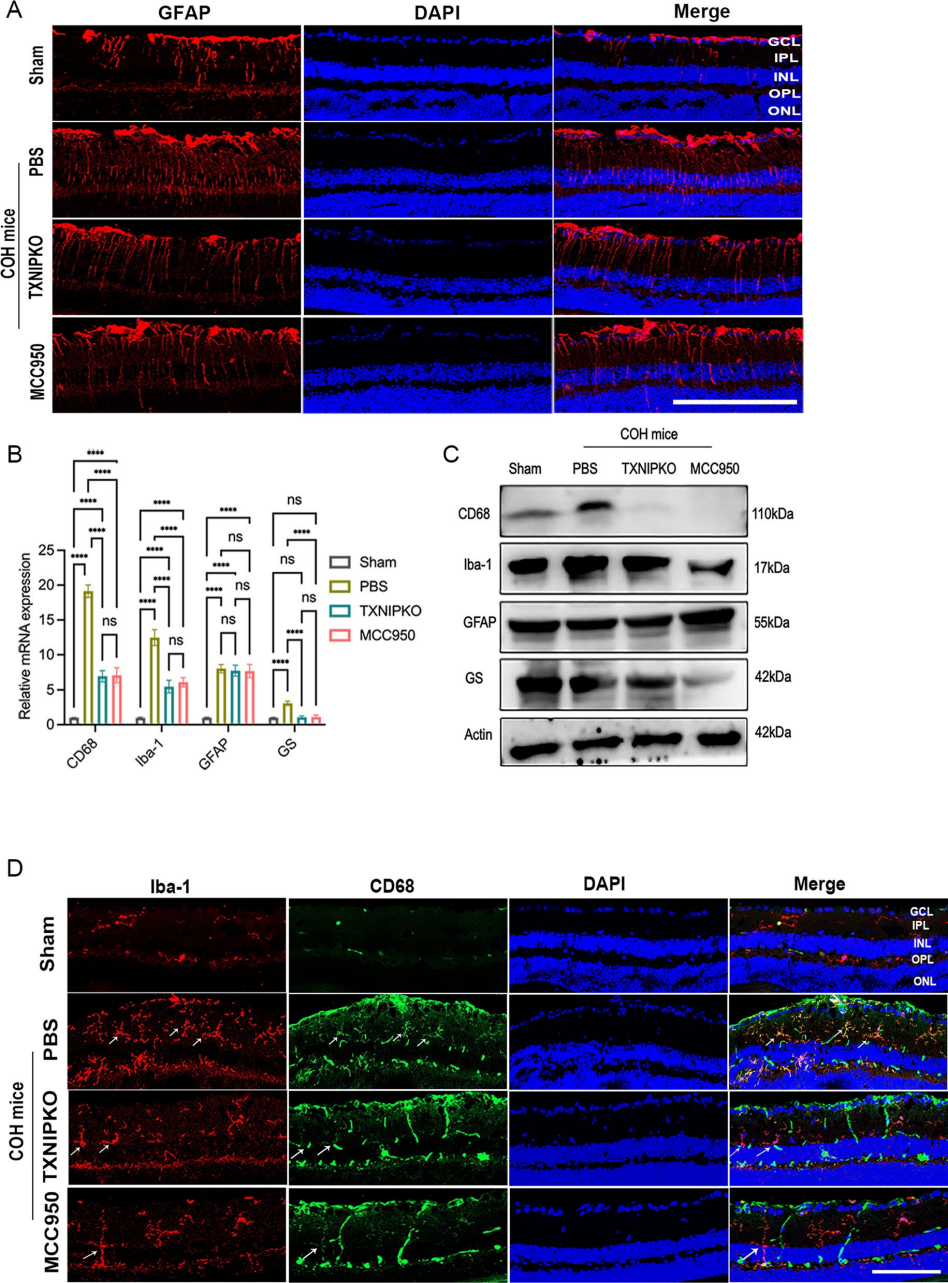
TXNIP mediated neuroinflammation in retina by activating microglia. **A** Immunofluorescent staining of GFAP (red) in retinal slices at 4 weeks of COH mice in different groups. **B**, **C** Western blotting and real-time RT-PCR analysis of markers of three glial at 4 weeks of COH in different groups. **D** Double immunofluorescent staining of CD68 and Iba-1 in retinal slices of COH mice in different groups (white arrows represent microglia that expressing CD68 and Iba-1) (magnification 200 × , scale bar = 50 μm). n = 8 per group for immunofluorescent staining, real-time RT–PCR, Western blotting. One-way ANOVA was performed. *p < 0.05, **p < 0.01, ***p < 0.001 and ****p < 0·0001, ns, no significance. Bars represent the mean ± SD. ONL, outer nuclear layer; OPL, outer plexiform layer; INL, inner nuclear layer; IPL, inner plexiform layer; GCL, ganglion cell layer

**Fig. 3:**
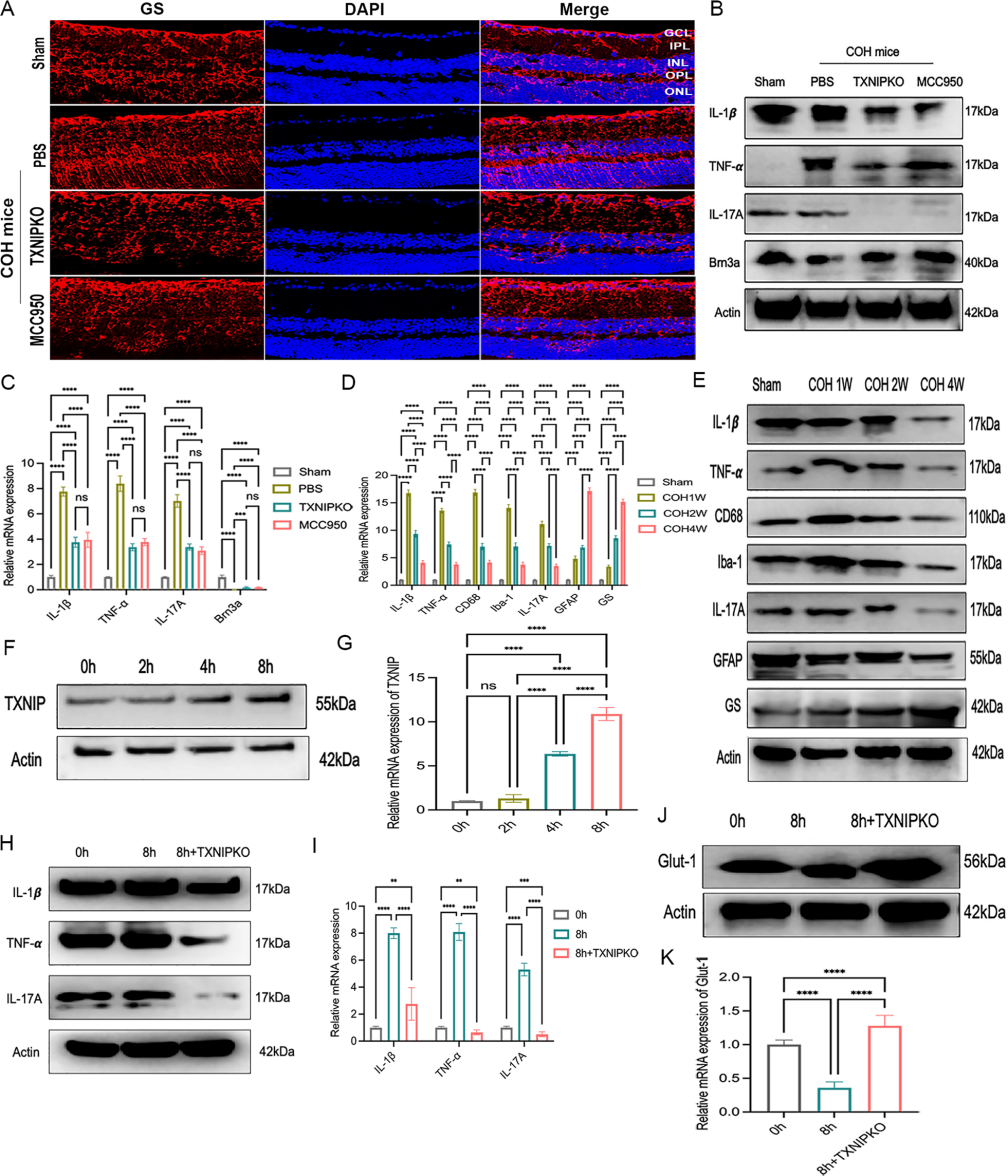
TXNIP silence suppressed proinflammatory factors secretion and facilitated M1 polarization of pressurized microglia in vitro. **A** Immunofluorescent staining of GS (red) in retinal slices at 4 weeks of COH mice in different groups. **B**, **C** Western blotting and real-time RT-PCR analysis of proinflammatory factors and Brn3a at 4 weeks of COH in different groups. **D**, **E** Western blotting and real-time RT-PCR analysis of proinflammatory factors and cell markers in TXNIP-deficiency COH mice. **F**–**G** Western blotting and real-time RT-PCR analysis of TXNIP in microglia cultured at 37.5 mmHg. **H**, **I** Western blotting and real-time RT-PCR analysis of proinflammatory factors in microglia of wild type and TXNIP-deficiency cultured at 37.5 mmHg for 8 h. **J**, **K** Western blotting and real-time RT-PCR analysis of the expression of Glut-1 in wild type or TXNIP-deficiency microglia cultured under normal or high pressure for 8 h. n = 8 per group for real-time RT–PCR and Western blotting. One-way ANOVA was performed. *p < 0.05, **p < 0.01, ***p < 0.001 and ****p < 0.0001, ns, no significance. Bars represent the mean ± SD

Three major classes of glial cells in the retina, namely, Müller cells, astrocytes and microglia, have all been reported to be profoundly involved in the occurrence and development of glaucoma. To determine which glial cells may be the predominant site of TXNIP action in glaucomatous conditions, we examined the spatiotemporal consistency of glial activation and retinal inflammation levels in TXNIP-deficient and MCC950-treated COH mice. Compared with PBS-treated COH mice, both MCC950-treated and TXNIP-deficient COH mice showed significantly decreased levels of glial fibrillary acidic protein (GFAP), glutamine synthetase (GS), CD68 and Iba-1 in the retinas (Figs. 2A, D, 3A, Supplementary Fig. 2B–D). The protein and mRNA expressions levels of CD68, Iba-1 and GS were markedly decreased in both TXNIP-deficient and MCC950-treated COH mice. However, no obvious difference in GFAP expression was observed between the TXNIP-deficient and MCC950-treated groups of COH mouse retinas (Fig. 2B, C, Supplementary Fig. 2A). As shown by the above results, the activation trends of retinal microglia and Müller cells were consistent in all tests in each experimental group.

To verify whether TXNIP is involved in the pathological mechanisms of experimental glaucoma by mediating microglial or Müller cell activation, we examined the temporal and spatial consistency of the dynamic secretion of inflammatory factors and the trend of glial activation in TXNIP-deficient COH mice. We found that ablation of TXNIP or blockade of the NLRP3 pathway observably reduced the release of proinflammatory cytokines, including TNF-α, IL-17A and IL-1β, in glaucomatous retinas (Fig. 3B, C, Supplementary Fig. 2E). In TXNIP-deficient mice, the expression of TNF-α, IL-17A, IL-1β, CD68 and Iba-1 peaked one week after modeling and decreased gradually as the duration of COH increased. However, the expression levels of GS and GFAP showed different tendencies during the process of experimental glaucoma. The mRNA expression levels of GS and GFAP increased with the extension of modeling time, while at the protein level, the expression levels of GS and GFAP showed completely opposite trends (Fig. 3D, E, Supplementary Fig. 2F). The above data indicated that only the trend of microglial activation was temporally and spatially consistent with the secretion of inflammatory factors in the retina in experimental glaucoma.

The results of in vitro experiments indicated that the expression levels of TXNIP increased in primary retinal microglia exposed to high pressure (Fig. 3F, G, Supplementary Fig. 2G). In addition, when we studied retinal microglia that were derived from TXNIP-deficient mice and cultured under high pressure for 8 h, we found that TXNIP gene knockout suppressed the secretion of proinflammatory factors compared with their secretion by wild-type microglia (Fig. 3H, I, Supplementary Fig. 2H). Taken together, our findings suggest that TXNIP probably mediates neuroinflammation in experimental glaucoma by activating retinal microglia.

### Silencing of TXNIP facilitated M1 microglial polarization under high pressure in vitro

**Fig. 4:**
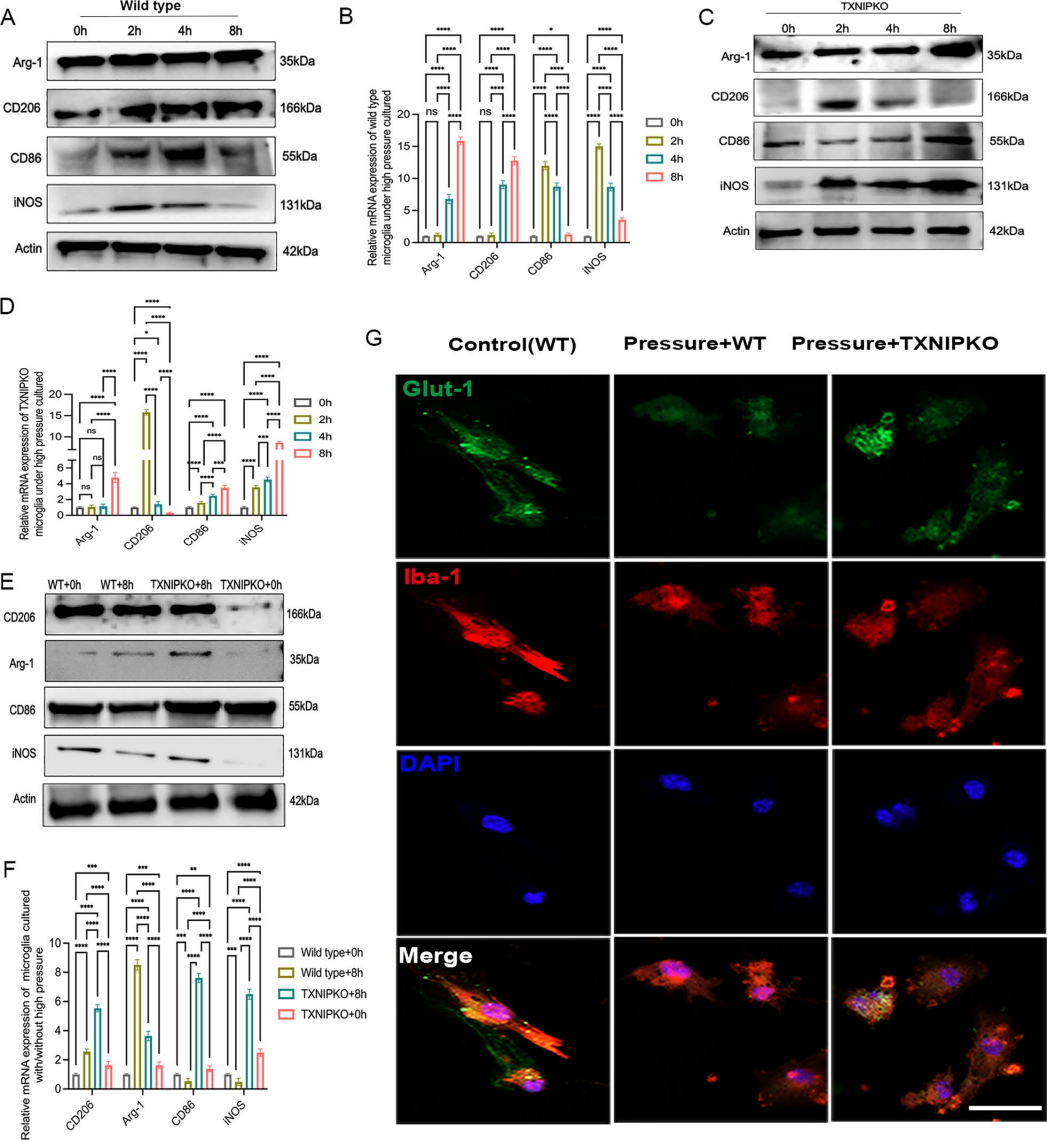
TXNIP silence facilitated M1 polarization of pressurized microglia in vitro (**A**). **A**, **B** Western blotting and real-time RT-PCR analysis of polarization markers of wild-type microglia cultured at 37.5 mmHg. **C**, **D** Western blotting and real-time RT-PCR analysis of polarization markers in TXNIP-deficiency microglia cultured at 37.5 mmHg. **E**, **F** Western blotting and real-time RT-PCR analysis of polarization markers of wild type and TXNIP-deficiency microglia cultured under normal pressure or at 37.5 mmHg for 8 h. **G** Immunofluorescent images showed the expression of Glut-1 in wild type or TXNIP-deficiency microglia cultured under normal or high pressure for 8 h (magnification 200 × , scale bar = 50 μm). n = 8 per group for immunofluorescent staining, real-time RT–PCR and Western blotting. One-way ANOVA was performed. *p < 0.05, **p < 0.01, ***p < 0.001 and ****p < 0.0001, ns, no significance. Bars represent the mean ± SD

**Fig. 5:**
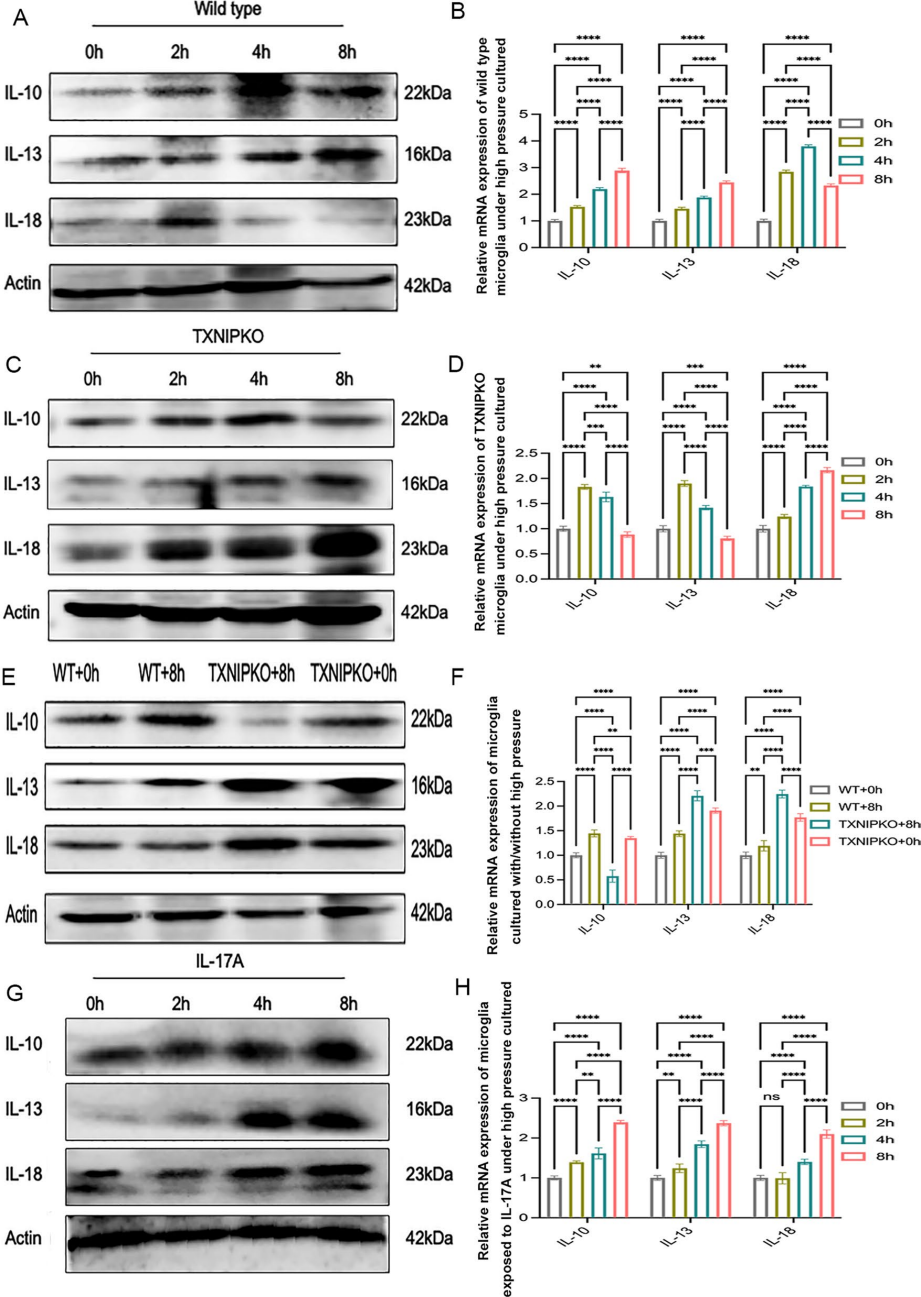
TXNIP silence facilitated M1 polarization of pressurized microglia in vitro (**B**). **A**, **B** Western blotting and real-time RT-PCR analysis of IL-10, IL-13, IL-18 of wild-type microglia cultured at 37.5 mmHg. **C**, **D** Western blotting and real-time RT-PCR analysis of IL-10, IL-13, IL-18 in TXNIP-deficiency microglia cultured at 37.5 mmHg. E-F. Western blotting and real-time RT-PCR analysis of IL-10, IL-13,IL-18 of wild type and TXNIP-deficiency microglia cultured at 37.5 mmHg for 8 h. **G**, **H** Western blotting and real-time RT-PCR analysis of M1/M2 cellular markers of microglia pretreated with rmIL-17A cultured at 37.5 mmHg. n = 8 per group for real-time RT–PCR, Western blotting. One-way ANOVA was performed. *p < 0.05, **p < 0.01, ***p < 0.001 and ****p < 0.0001, ns, no significance. Bars represent the mean ± SD

We next analyzed the relationship between TXNIP and microglial phenotype transformation in experimental glaucoma. The expression of M1-like microglial markers (CD86, iNOS, IL-18) and M2-like microglial markers (Arg-1, CD206, IL-13, IL-10) was detected in high-pressure culture. In the process of pressurized culturing, the trend of transformation to the M2-like phenotype was enhanced in wild-type microglia, while the trend towards the M1-like phenotype was weakened (Fig. 4A, B, Supplementary Fig. 2I, Fig. 5A, B, Supplementary Fig. 2M). Compared with wild-type microglia, TXNIP-deficient microglia showed an enhanced trend of transformation to the M1-like phenotype, whereas silencing TXNIP did not have a significant influence on M2-like microglial polarization (Fig. 4C–F, Supplementary Fig. 2J,K, Fig. 5C–F, Supplementary Fig. 2N, O). These results indicate that TXNIP is mainly involved in the negative regulation of M1-like microglial transformation and has little effect on M2-like microglial polarization under high pressure. Given that TXNIP mediates energy metabolism by regulating glucose uptake and that remodeling of energy metabolism is found in the process of changing microglial polarity, we explored the variation in glucose transporter-1(Glut-1) levels in microglia in the context of TXNIP knockout in an experimental glaucoma scenario. The results showed that ablation of TXNIP promoted the expression of Glut-1 in microglia under high-pressure culture (Fig. 2J, K, Supplementary Fig. 2L, Fig. 4G). These data suggested that TXNIP was probably involved in regulating the phenotypic transformation of retinal microglia under the pathological condition of high pressure.

### TXNIP and IL-17A synergistically participated in the regulation of retinal microglial phenotypic transformation

**Fig. 6:**
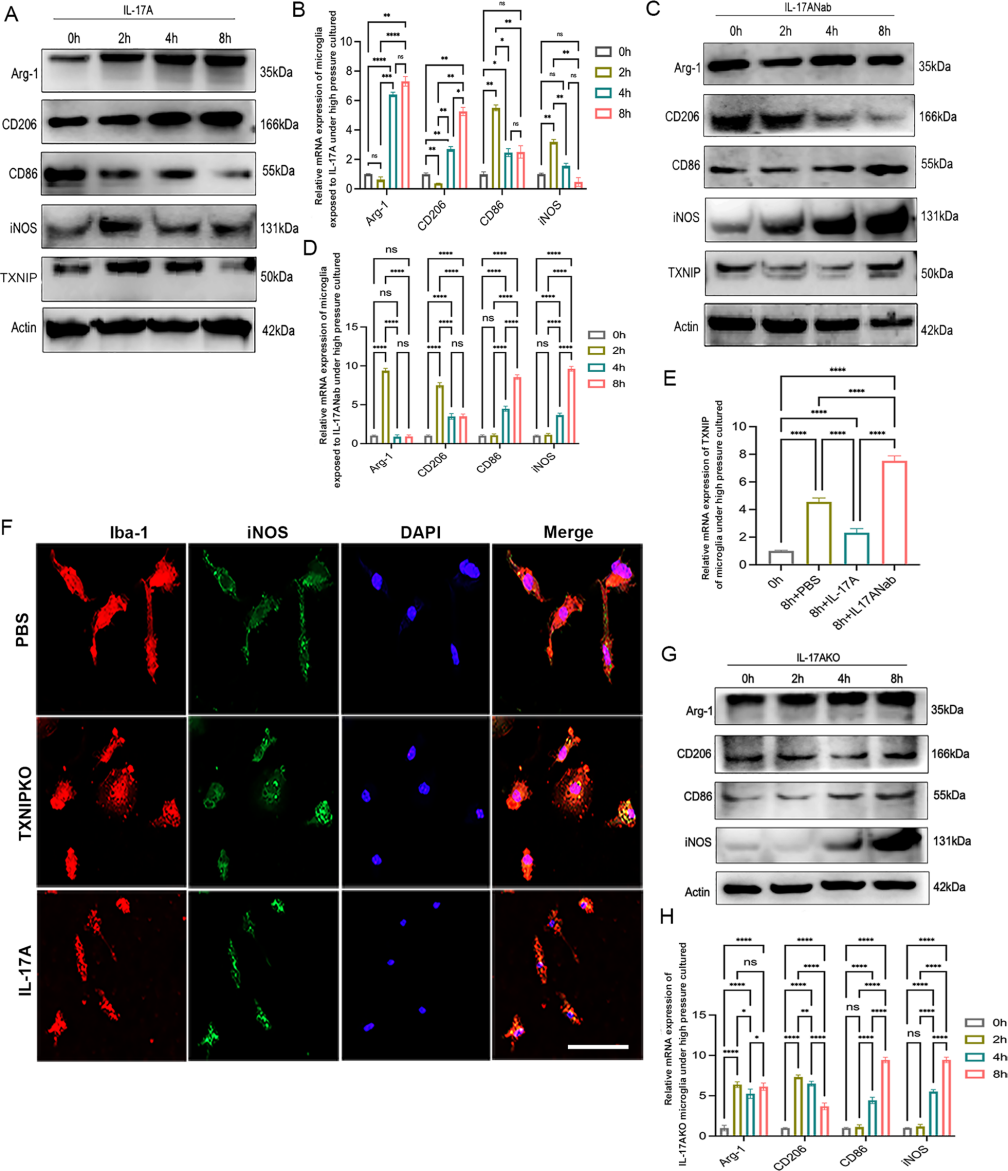
TXNIP and IL-17A synergistically participated in the regulation of retinal microglial phenotype transformation. **A**, **B** Western blotting and real-time RT-PCR analysis of M1/M2 cellular markers and TXNIP of microglia pretreated with rmIL-17A cultured at 37.5 mmHg. **C**, **D** Western blotting and real-time RT-PCR analysis of M1/M2 cell markers and TXNIP of microglia pretreated with IL-17ANab cultured at 37.5 mmHg. **E** Real-time RT-PCR analysis of TXNIP in microglia cultured at 37.5 mmHg. **F** Double immunofluorescent staining of iNOS (green) and Iba-1(red) in wild type or TXNIP-deficiency microglia pretreated with rmIL-17A under normal pressure cultured (magnification 200 × , scale bar = 50μm). **G**, **H** Western blotting and real-time RT-PCR analysis of M1/M2 cellular markers in IL-17A-deficiency microglia cultured at 37.5 mmHg. n = 8 per group for immunofluorescent staining, real-time RT–PCR and Western blotting. One-way ANOVA was performed. *p < 0.05, **p < 0.01, ***p < 0.001 and ****p < 0.0001, ns, no significance. Bars represent the mean ± SD

**Fig. 7:**
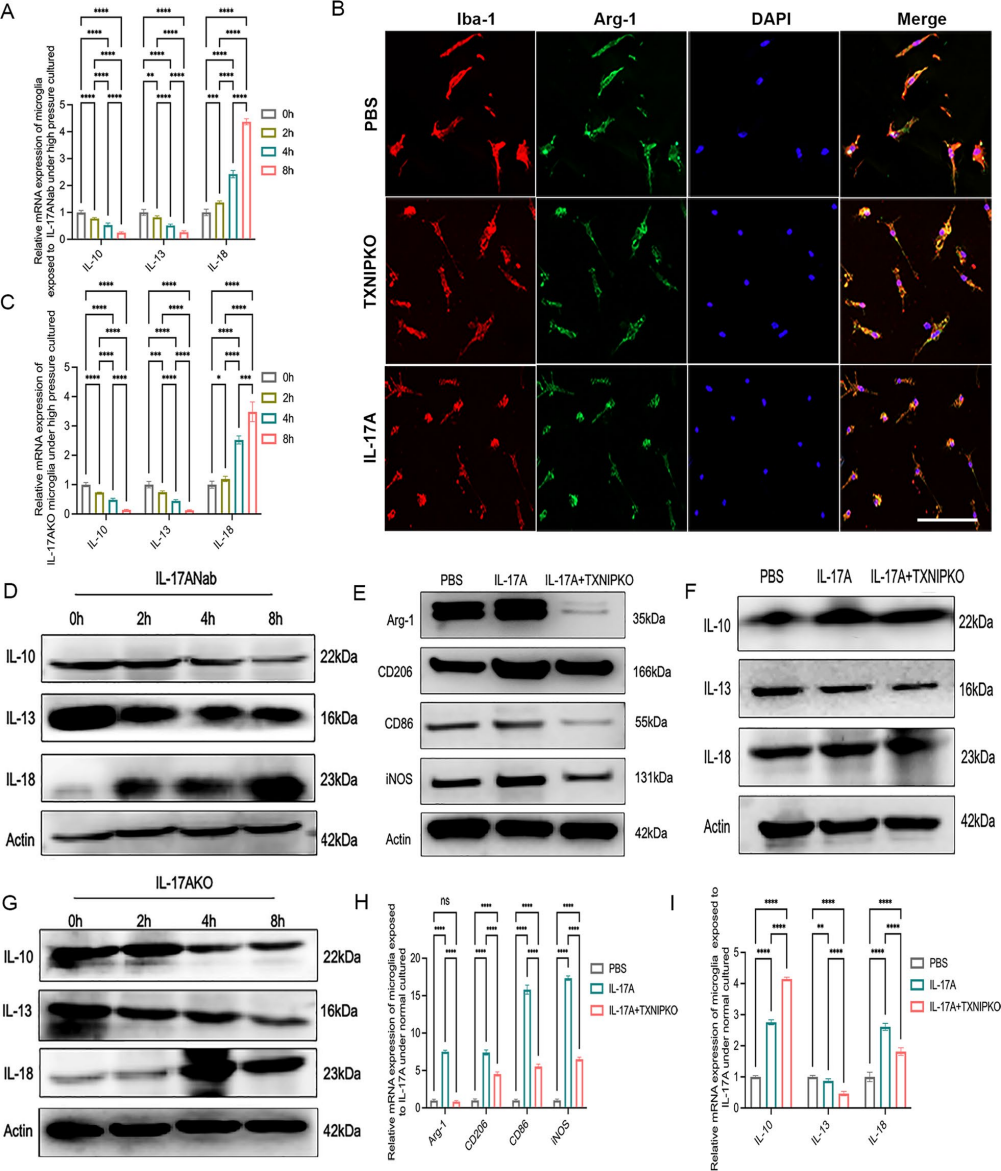
TXNIP and IL-17A synergistically participated in the regulation of retinal M1/M2 microglial transformation. **A**, **D** Western blotting analysis and real-time RT-PCR analysis of expression of IL-10, IL-13 and IL-18 of microglia pretreated with IL-17ANab cultured at 37.5 mmHg. **B** Double immunofluorescent staining of Arg-1 (green) and Iba-1(red) in wild type or TXNIP-deficiency microglia pretreated with rmIL-17A under normal pressure cultured (magnification 200 × , scale bar = 50 μm). **C**, **G** Western blotting analysis and real-time RT-PCR analysis of IL-10, IL-13 and IL-18 in IL-17A-deficiency microglia cultured at 37.5 mmHg. **E**, **H** Western blotting analysis and real-time RT-PCR analysis of M1/M2 cellular markers in wild type or TXNIP-deficiency microglia pretreated with rmIL-17A under normal pressure cultured. **F**, **I** Western blotting analysis and real-time RT-PCR analysis of IL-10, IL-13 and IL-18 in wild type or TXNIP-deficiency microglia pretreated with rmIL-17A under normal pressure cultured. n = 8 per group for immunofluorescent staining, real-time RT–PCR, Western blotting. One-way ANOVA was performed. *p < 0.05, **p < 0.01, ***p < 0.001 and ****p < 0·0001, ns, no significance. Bars represent the mean ± SD

In our previous study, we found that IL-17A was dynamically involved in the regulation of retinal microglial phenotypic transformation, which was associated with the duration of IOP elevation (Chen et al. 2023). In this work, we verified the impact of IL-17A on the phenotypic alteration of microglia in vitro. The results showed that the phenotypic changes in microglia exposed to high pressure with rmIL-17A pretreatment gradually shifted towards M2-like polarization, while the trend of transformation to M1-like cells was weakened (Fig. 6A, B, Supplementary Fig. 3B). In Fig. 5G–H (Supplementary Fig. 3A), the trend of IL-18 expression was opposed to those of iNOS and CD86 expression, while the trends of IL-10 and IL-13 were consistent with those of Arg-1 and CD206. When the expression of IL-17A was suppressed by IL-17A Nab or gene knockout, the trend of microglial phenotypic transformation to an M1-like phenotype was reversed during pressurized culture (Fig. 6C, D, G, H; Supplementary Fig. 3C–F; Fig. 7A, C, D, G). Since the polarization transformation of microglia is accompanied by a shift in the cellular energy production pattern from oxidative phosphorylation to aerobic glycolysis, in which TXNIP is essential, we hypothesized that IL-17A mediated the phenotypic change in retinal microglia in close association with TXNIP. First, we found that rmIL-17A pretreatment, compared with PBS treatment, reduced the expression of TXNIP in pressurized microglia (Fig. 6A, B, Supplementary Fig. 3B), whereas stimulation with IL-17A Nab restored the increased expression of TXNIP induced by pressurization (Fig. 6C, E, Supplementary Fig. 3C). Afterwards, rmIL-17A intervention was performed on microglia cultured under normal pressure. Compared to wild-type microglia, the immunofluorescence analysis illustrated markedly reduced expression of iNOS and increased expression of Arg-1 in TXNIP-deficient microglia (Figs. 6F, 7B). Western blotting and RT-PCR results showed that the transformation of the microglial phenotype to M1-like cells was inhibited in TXNIP-deficient microglia compared with wild-type microglia when both groups were exposed to rmIL-17A for 24 h (Fig. 7E, F, H, I; Supplementary Fig. 3G–H). Taken together, our data indicated that IL-17A might act synergistically with TXNIP to participate in regulating the phenotypic transformation of retinal microglia.

### TXNIP regulated microglial phenotypic transformation via the PI3K/Akt signaling pathway in experimental glaucoma

**Fig. 8:**
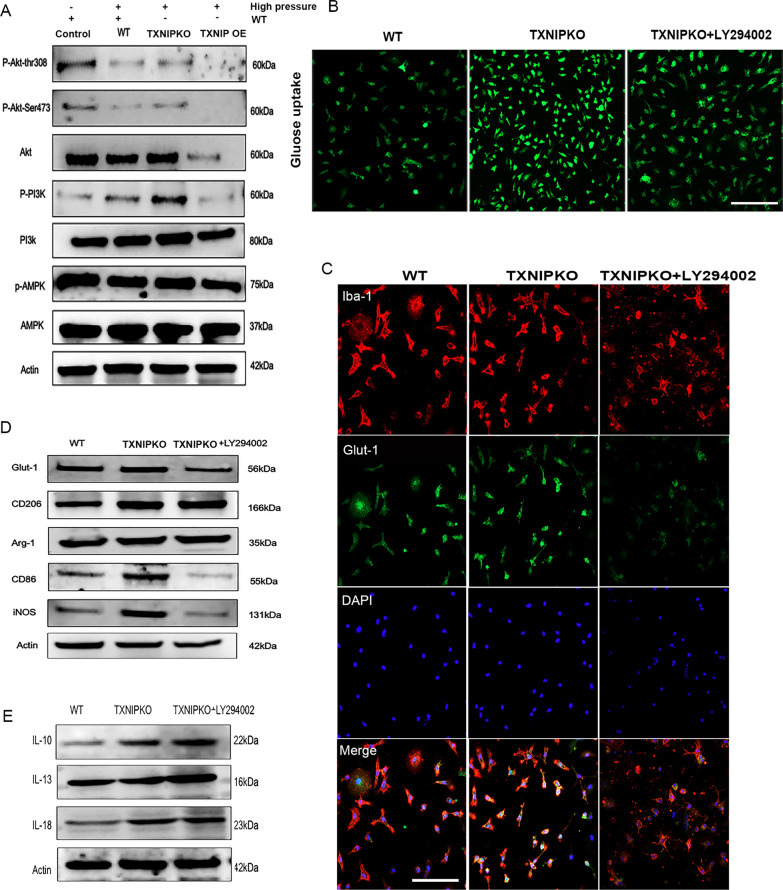
TXNIP regulated M1-like microglia transformation via the PI3K/Akt signaling pathway in experimental glaucoma. **A** Western blotting analysis of the expression of AMPK and PI3K/Akt signaling pathway proteins in microglia from wildtype or distinctive TXNIP-types cultured at 37.5 mmHg. **B** Glucose uptake of microglia cultured at 37.5 mmHg. **C** Double immunofluorescent staining of Glut-1 (green) and Iba-1(red) in TXNIP-deficiency microglia pretreated with or without LY294002 cultured at 37.5 mmHg (magnification 100 × , scale bar = 100 μm). **D** Western blotting analysis of the expression of Glut-1 and M1/M2 cellular markers in TXNIP-deficiency microglia pretreated with or without LY294002 cultured at 37.5 mmHg. **E** Western blotting analysis of the expression of IL-10, IL-13 and IL-18 in TXNIP-deficiency microglia pretreated with or without LY294002 cultured at 37.5 mmHg. n = 8 per group for immunofluorescent staining and Western blotting. One-way ANOVA was performed. *p < 0.05, **p < 0.01, ***p < 0.001 and ****p < 0.0001, ns, no significance. Bars represent the mean ± SD

The AMPK and PI3K/Akt signaling pathways are two of the classical pathways regulating energy metabolism and are reported to be related to the expression or regulatory function of TXNIP (Xu et al. 2019). Therefore, to explore the specific mechanism underlying the regulation of retinal microglial phenotypic transformation by TXNIP in experimental glaucoma, we screened and verified these two signaling pathways in vitro. Compared with those in wild-type microglia, the phosphorylation levels of PI3K and Akt increased significantly in TXNIP-deficient microglia under high pressure in vitro, while no obvious change in AMPK phosphorylation was observed. In addition, the phosphorylation of PI3K, Akt and AMPK was significantly reduced after TXNIP overexpression under culture conditions similar to those described above (Fig. 8A, Supplementary Fig. 3K). Hence, we speculated that TXNIP might affect the stress reaction of microglia through the PI3K/Akt signaling pathway in experimental glaucoma. Following the above results we used the same experimental scenario to assess microglial polarity, glucose uptake and Glut-1 expression after pretreatment with the PI3K inhibitor LY294002. We found that the expression levels of CD86, iNOS, IL-18 and Glut-1 as well as glucose uptake were significantly reduced in TXNIP-deficient microglia pretreated with LY294002 (Fig. 8B–E, Supplementary Fig. 3I, J), indicating that TXNIP probably regulated the M1-like polarization of microglia and glucose uptake through the PI3K/Akt signaling pathway.

## Discussion

Despite abundant emerging data on neuroprotection in glaucoma, the discovery of endogenous targets that contribute to the prevention or restoration of glaucomatous neurodegeneration is lacking. In the present study, we demonstrated that TXNIP, a key regulator of energy metabolism, has a promising role as an endogenous target for neuroprotective interventions in experimental glaucoma neuropathy regarding the regulation of neuroinflammation and microglial activation.

We showed that with the prolongation of COH, retinal TXNIP was upregulated and mainly expressed in the GCL. This phenomenon was consistent with the report of Munemasa et al. (2009), which indicated that increased retinal expression of TXNIP was observed at both 2 and 5 weeks after glaucoma modeling. Moreover, we also focused on TXNIP function in the neuropathy process and found that deletion of TXNIP enhanced RGC survival and preserved retinal neuronal function in experimental glaucoma. Our result complemented those of a previous study implying that RGC death was prevented in the absence of TXNIP under retinal neurodegeneration, indicating the possible prospects of TXNIP as a new endogenous therapeutic target for neuroprotection in glaucoma (Ao et al. 2021). Furthermore, TXNIP was identified as an activator of the NLRP3 inflammasome. Therefore, we also explored the degree to which visual function was retained after NLRP3 was blocked by MCC950. The results showed that the impact of TXNIP, which exacerbated the glaucomatous neurodegeneration, was partly dependent on the activation of NLRP3. Previous studies have found that NLRP3 and TXNIP may regulate neuroinflammation in the CNS and retina through the toll-like-receptor-4 (TLR4) and P2X7 purine receptors (P2X7R) pathways. In the PD model, TLR4 silencing decreased the expression levels of NLRP3, TXNIP, IL-1β and IL-18, while activation of TLR4 in microglia promoted the expression of IL-1β and IL-18 (Zhang et al. 2020). In diabetic retinopathy, the NLRP3 inhibitor verapamil can significantly inhibit TLR4-mediated NLRP3 activation, reduce IL-1β and TNF-α levels and improve RGC survival (Eissa et al. 2021). It has been reported that neuroinflammation is actively related to P2X7R activation by risk-associated molecular patterns (DAMPs), of which is extracellular ATP is the chief example. In the retina, P2X7R is expressed on Müller cells, vascular endothelial cells, microglia and other cells. Stimulating P2X7R signaling activates NLRP3 and promotes IL-1β maturation and release, exacerbating inflammation and cell death (Kong et al. 2022). In addition, inhibiting P2X7R in microglia can indirectly reduce NLRP3 activity (Franceschini et al. 2015). We speculate that in our study, the protective effect of TXNIP and NLRP3 ablation on RGCs may be related to the inhibition of the TLR4 and P2X7R pathways in microglia, which would reduce the level of neuroinflammation. However, whether the neuroprotection introduced by TXNIP-deficiency is due only to the inhibition of the retinal inflammatory response or is also attributed to a combined synergistic effect resulting from TXNIP-related changes in RGC metabolism requires further investigation.

Our data further suggested that TXNIP was involved in the regulation of neuroinflammation, probably by activating microglia in the glaucomatous retina. Although the activation levels of the three distinct types of retinal glial cells decreased to varying degrees after TXNIP ablation, only the activation of microglia presented temporal and spatial consistency with the rhythm of inflammatory factor secretion. In a retinal damage model induced by ischemia/reperfusion or hyperglycemia, TXNIP might influence Müller cell activation by regulating mitochondrial function (Munemasa et al. 2009; El-Azab et al. 2014). However, the relationship between TXNIP and astrocytes or microglia in retinal neurodegenerative diseases has not been clarified. The pathological role of TXNIP was interpreted as regulating microglial activation in experimental glaucoma in our study. The results indicated that the inflammatory responses of microglia to high-pressure stimulation both in vivo and in vitro were reduced with TXNIP elimination. Hence, TXNIP shows promise as an endogenous target through which to regulate microglial activation in glaucoma.

Our second major finding was that TXNIP mediated the phenotypic change in microglia in experimental glaucoma. Blockade TXNIP under pathological high-pressure conditions increased the tendency of microglia to transform into M1-like cells. As highly tissue-specific macrophages, microglia assume a diverse range of phenotypes and have the ability to shift between distinctive phenotypes in response to specific stimuli to maintain tissue homeostasis. Increasing evidence suggests that microglial polarization to M1-like cells occurs along with metabolic reprogramming, which is the shift from oxidative phosphorylation to aerobic glycolysis (Devi et al. 2017; Coucha et al. 2019). Everts et al. reported that LPS-induced BV-2 polarization consisted of two metabolic steps, where the first step was characterized by increased intracellular glucose metabolism. As an important regulator of glucose and lipid metabolism, TXNIP promotes glucose uptake by enhancing Glut-1 expression (Tsubaki et al. 2020). In our study, we found that inhibition of TXNIP promoted the expression of Glut-1 in microglia in vivo. Therefore, we hypothesized that TXNIP might influence the polarization of microglia by regulating glucose metabolism; however, the specific underlying mechanism needs to be further explored.

Under physiological conditions, microglia retain an immune surveillance phenotype for constantly monitoring their surroundings; however, they switch to various states of activation when the tissue is injured or stressed (Orihuela et al. 2016). In our study, we found that activation of retinal microglia dynamically altered phenotype polarization in experimental glaucoma, which was similar to previous reported results in COH mice and oxygen-induced retinopathy models (Graeber 2010; Campagno et al. 2021). The present study also demonstrated that the polarization tendency of activated microglia changed in response to stimuli other than pressurization. Our data indicated that IL-17A acted synergistically with TXNIP to participate in the phenotypic regulation of retinal microglia. Under normal culture conditions, TXNIP elimination reduced the tendency of IL-17A-treated microglia to convert to M1-like cells. These results confirmed that the outcome of M1/M2 polarization was associated with various factors, including IL-17A and pressurization, as demonstrated in the present study, as well as environment stress; production of reactive oxygen species (ROS), iNOS, and proinflammatory factors; and activation of NLRP3, as illustrated in previous reports by others (Hu et al. 2021; Zhou et al. 2017; Li et al. 2021). Additionally, our results confirmed that microglia expressed both M1- and M2-like markers simultaneously, suggesting that there was a dynamic balance of the microglial polarization state between the two contrasting phenotypes in experimental glaucoma. Mechanistically, IL-17A may stimulate microglia to transform to a certain phenotype with a neuroprotective effect by regulating the immune response of cells, while TXNIP may contribute to the modulation of microglial phenotype switching under specific conditions by regulating the metabolism of microglia. TXNIP may also be involved in this process by regulating the microglial immune response in synergy with the impact of IL-17A in the early stage of experimental glaucoma. The specific underlying mechanisms need to be further explored.

Glaucoma is a heterogeneous disease involving complex mechanisms communication between different cell types as well as between cells and the microenvironment; these mechanisms have not been fully elucidated. In our study, TXNIP knockout was shown to have a protective effect against RGCs in vivo, while the silencing of TXNIP promoted proinflammatory M1 microglial polarization in vitro. We speculate that the internal mechanism of the above results may be as follows. First, our previous study found that the phenotypes of microglia were in a dynamic state during pathological hypertension, with the proportions of M1 and M2 microglia being related to the duration and degree of elevated IOP (Chen et al. 2023). The number of RGCs was counted at 4 weeks after COH, which was in the stable maintenance period of IOP in our model (Chen et al. 2023), whereas the current study used a short period (8 h) of high pressure in vitro. Therefore, the effect of TXNIP on the microglial phenotype may vary depending on the duration of high pressure. In addition, the protective effect of TXNIP knockout on RGCs in vivo may be a result of many factors, such as microglia, astrocytes, Müller cells and the cell microenvironment. The role of TXNIP in experimental glaucoma may vary and may be related to the time, frequency and degree of sustained high-pressure maintenance. Therefore, further research is needed to clarify the underlying relationship.

Finally, our data suggested that TXNIP mediated M1-like microglial polarization via the PI3K/Akt signaling pathway. We showed that a PI3K/Akt inhibitor reversed the trend of M1-type transformation of microglia due to TXNIP knockout. The PI3K/Akt signaling pathway is an active participant in biological energy metabolism and cellular behaviors and was reported to be involved in the occurrence and development of neurodegenerative diseases (Bordt and Polster 2014). In diabetic retinopathy, TXNIP was reported to regulate the autophagy and apoptosis of Müller cells via the PI3K/Akt/mTOR signaling pathway (Xu et al. 2019). To the best of our knowledge, we are the first to report that TXNIP affects microglial polarity transformation and glucose uptake through the PI3K/Akt pathway in experimental glaucoma. Regarding the mechanism, we hypothesized that TXNIP ablation regulated cellular energy metabolism by promoting the activation of the PI3K/Akt signaling pathway, thus facilitating microglial transformation toward the M1-like phenotype in a high-pressure environment. This hypothesis needs further investigation.

## Conclusions

In summary, our data suggest that TXNIP participates in the pathogenesis of glaucoma neuropathy and facilitates the polarization of retinal microglia to an M1-like phenotype via the PI3K/Akt signaling pathway. Our study demonstrates the beneficial role of TXNIP inhibition in the context of glaucomatous neurodegeneration and provides a theoretical basis for new breakthroughs in the field of glaucoma treatment.

## Supplementary Information

Additional file 1: Figure S1. A Statistical analysis of Western blotting analysis of TXNIP at 0, 2,4, and 8 weeks of COH. B Statistical analysis of immunofluorescent staining of Brn3a in retinal whole mounts at 4 weeks of COH mice in different groups. C, D Statistical analysis of F-VEP test at 4 weeks after COH modeling in different groups. E Statistical analysis of immunofluorescent staining of NEFH in retinal whole mounts at 4 weeks of COH mice in different groups. F Statistical analysis of IOP among 3 groups at different time points.

Additional file 2: Figure S2. A Statistical analysis of Western blotting analysis of markers of three glial at 4 weeks of COH in different groups. B, C Statistical analysis of immunofluorescent staining of GFAP and GS in retinal slices at 4 weeks of COH mice in different groups. D Statistical analysis of double immunofluorescent staining of CD68 and Iba-1 in retinal slices of COH mice in different groups. E Statistical analysis of Western blotting analysis of proinflammatory factors and Brn3a at 4 weeks of COH in different groups. F Statistical analysis of Western blotting analysis of proinflammatory factors and cell markers in TXNIP-deficiency COH mice. G Statistical analysis of Western blotting analysis of TXNIP in microglia cultured at 37.5 mmHg. H Statistical analysis of Western blotting analysis of proinflammatory factors in microglia of wild type and TXNIP-deficiency cultured at 37.5 mmHg for 8 h. I Statistical analysis of Western blotting analysis of polarization markers of wild-type microglia cultured at 37.5 mmHg. J Statistical analysis of Western blotting analysis of polarization markers in TXNIP-deficiency microglia cultured at 37.5 mmHg. K Statistical analysis of Western blotting analysis of polarization markers of wild type and TXNIP-deficiency microglia cultured under normal pressure or at 37.5 mmHg for 8 h. L Statistical analysis of Western blotting analysis of the expression of Glut-1 in wild type or TXNIP-deficiency microglia cultured under normal or high pressure for 8 h. M Statistical analysis of Western blotting analysis of IL-10, IL-13, IL-18 of wild-type microglia cultured at 37.5 mmHg. N Statistical analysis of Western blotting analysis of IL-10, IL-13 and IL-18 in TXNIP-deficiency microglia cultured at 37.5 mmHg. O Statistical analysis of Western blotting analysis of IL-10, IL-13 and IL-18 of wild type and TXNIP-deficiency microglia cultured at 37.5 mmHg for 8 h.

Additional file 3: Figure S3. A Statistical analysis of Western blotting analysis of IL-10, IL-13 and IL-18 of microglia pretreated with rmIL-17A cultured at 37.5 mmHg. B Statistical analysis of Western blotting analysis of M1/M2 cellular markers and TXNIP of microglia pretreated with rmIL-17A cultured at 37.5 mmHg. C Statistical analysis of Western blotting analysis of M1/M2 cell markers of microglia pretreated with IL-17ANab cultured at 37.5 mmHg. D Statistical analysis of Western blotting analysis of M1/M2 cellular markers in IL-17A-deficiency microglia cultured at 37.5 mmHg. E Statistical analysis of Western blotting analysis of IL-10, IL-13 and IL-18 of microglia pretreated with IL-17ANab cultured at 37.5 mmHg. F Statistical analysis of Western blotting analysis of IL-10, IL-13 and IL-18 in IL-17A-deficiency microglia cultured at 37.5 mmHg. G Statistical analysis of Western blotting analysis of M1/M2 cellular markers in wild type or TXNIP-deficiency microglia pretreated with rmIL-17A under normal pressure cultured. H Statistical analysis of Western blotting analysis of IL-10, IL-13 and IL-18 in wild type or TXNIP-deficiency microglia pretreated with rmIL-17A under normal pressure cultured. I Statistical analysis of Western blotting analysis of the expression of Glut-1 and M1/M2 cellular markers in TXNIP-deficiency microglia pretreated with or without LY294002 cultured at 37.5 mmHg. J Statistical analysis of Western blotting analysis of the expression of IL-10, IL-13 and IL-18 in TXNIP-deficiency microglia pretreated with or without LY294002 cultured at 37.5 mmHg. K Statistical analysis of Western blotting analysis of the expression of AMPK and PI3K/Akt signaling pathway proteins in microglia from wildtype or distinctive TXNIP-types cultured at 37.5 mmHg.

## Data Availability

The datasets used and analysed during the current study are available from the corresponding author on reasonable request.

## Abbreviations

TXNIP: Thioredoxin-interacting protein
RGCs: Retinal ganglion cells
CNS: Central nervous system
RPE: Retinal pigment epithelium
NLRP3: Thermo-containing protein domain 3
COH: Chronic ocular hypertension
KO: Knockout
PBS: Phosphate-buffered saline
IOP: Intraocular pressure
FBS: Fetal bovine serum
ERK1/2: Extracellular signal-regulated kinase 1/2
F-VEP: Flash visual-evoked potential
PFA: Paraformaldehyde
OCT: Optimum cutting temperature compound
DEGs: Differentially expressed genes
Glut-1: Glucose transporter-1
GS: Glutamine synthetase

## References

[CR1] Agarwal R, Agarwal P. Rodent models of glaucoma and their applicability for drug discovery. Expert Opin Drug Discov. 2017;12(3):261–70. 28075618 10.1080/17460441.2017.1281244

[CR2] Ao H, Li H, Zhao X, Liu B, Lu L. TXNIP positively regulates the autophagy and apoptosis in the rat müller cell of diabetic retinopathy. Life Sci. 2021;267: 118988. 33412212 10.1016/j.lfs.2020.118988

[CR3] Baudouin C, Kolko M, Melik-Parsadaniantz S, Messmer EM. Inflammation in glaucoma: from the back to the front of the eye, and beyond. Prog Retin Eye Res. 2021;83: 100916. 33075485 10.1016/j.preteyeres.2020.100916

[CR4] Bordt EA, Polster BM. NADPH oxidase- and mitochondria-derived reactive oxygen species in proinflammatory microglial activation: a bipartisan affair? Free Radic Biol Med. 2014;76:34–46. 25091898 10.1016/j.freeradbiomed.2014.07.033 PMC4252610

[CR5] Campagno KE, Lu W, Jassim AH, Albalawi F, Cenaj A, Tso HY, et al. Rapid morphologic changes to microglial cells and upregulation of mixed microglial activation state markers induced by P2X7 receptor stimulation and increased intraocular pressure. J Neuroinflammation. 2021;18(1):217. 34544431 10.1186/s12974-021-02251-7 PMC8454080

[CR6] Chen J, Sun J, Yu H, Huang P, Zhong Y. Evaluation of the effectiveness of a chronic ocular hypertension mouse model induced by intracameral injection of cross-linking hydrogel. Front Med (Lausanne). 2021;8:643402. 33829024 10.3389/fmed.2021.643402 PMC8019751

[CR7] Chen J, Zhong H, Yu H, Sun J, Shen B, Xu X, et al. Interleukin-17A modulates retinal inflammation by regulating microglial activation via the p38 MAPK pathway in experimental glaucoma neuropathy. Faseb j. 2023;37(6): e22945. 37144630 10.1096/fj.202202056RR

[CR8] Coucha M, Shanab AY, Sayed M, Vazdarjanova A, El-Remessy AB. Modulating expression of thioredoxin interacting protein (TXNIP) prevents secondary damage and preserves visual function in a mouse model of ischemia/reperfusion. Int J Mol Sci. 2019;20(16):3969. 31443163 10.3390/ijms20163969 PMC6721134

[CR9] Devi TS, Somayajulu M, Kowluru RA, Singh LP. TXNIP regulates mitophagy in retinal Müller cells under high-glucose conditions: implications for diabetic retinopathy. Cell Death Dis. 2017;8(5): e2777. 28492550 10.1038/cddis.2017.190 PMC5520711

[CR10] Devi TS, Yumnamcha T, Yao F, Somayajulu M, Kowluru RA, Singh LP. TXNIP mediates high glucose-induced mitophagic flux and lysosome enlargement in human retinal pigment epithelial cells. Biol Open. 2019;8(4):bio038521. 31023645 10.1242/bio.038521 PMC6503994

[CR11] Eissa LD, Ghobashy WA, El-Azab MF. Inhibition of thioredoxin-interacting protein and inflammasome assembly using verapamil mitigates diabetic retinopathy and pancreatic injury. Eur J Pharmacol. 2021;901: 174061. 33766618 10.1016/j.ejphar.2021.174061

[CR12] El-Azab MF, Baldowski BR, Mysona BA, Shanab AY, Mohamed IN, Abdelsaid MA, et al. Deletion of thioredoxin-interacting protein preserves retinal neuronal function by preventing inflammation and vascular injury. Br J Pharmacol. 2014;171(5):1299–313. 24283717 10.1111/bph.12535 PMC3952806

[CR13] Franceschini A, Capece M, Chiozzi P, Falzoni S, Sanz JM, Sarti AC, et al. The P2X7 receptor directly interacts with the NLRP3 inflammasome scaffold protein. Faseb J. 2015;29(6):2450–61. 25690658 10.1096/fj.14-268714

[CR14] Graeber MB. Changing face of microglia. Science. 2010;330(6005):783–8. 21051630 10.1126/science.1190929

[CR15] Hu X, Zhao GL, Xu MX, Zhou H, Li F, Miao Y, et al. Interplay between Müller cells and microglia aggravates retinal inflammatory response in experimental glaucoma. J Neuroinflammation. 2021;18(1):303. 34952606 10.1186/s12974-021-02366-x PMC8705189

[CR16] Kong H, Zhao H, Chen T, Song Y, Cui Y. Targeted P2X7/NLRP3 signaling pathway against inflammation, apoptosis, and pyroptosis of retinal endothelial cells in diabetic retinopathy. Cell Death Dis. 2022;13(4):336. 35410316 10.1038/s41419-022-04786-w PMC9001662

[CR17] Li J, Yu S, Lu X, Cui K, Tang X, Xu Y, et al. The phase changes of M1/M2 phenotype of microglia/macrophage following oxygen-induced retinopathy in mice. Inflamm Res. 2021;70(2):183–92. 33386422 10.1007/s00011-020-01427-w

[CR18] Munemasa Y, Ahn JH, Kwong JM, Caprioli J, Piri N. Redox proteins thioredoxin 1 and thioredoxin 2 support retinal ganglion cell survival in experimental glaucoma. Gene Ther. 2009;16(1):17–25. 18701913 10.1038/gt.2008.126

[CR19] O’Koren EG, Yu C, Klingeborn M, Wong AYW, Prigge CL, Mathew R, et al. Microglial function is distinct in different anatomical locations during retinal homeostasis and degeneration. Immunity. 2019;50(3):723-737.e727. 30850344 10.1016/j.immuni.2019.02.007 PMC6592635

[CR20] Orihuela R, McPherson CA, Harry GJ. Microglial M1/M2 polarization and metabolic states. Br J Pharmacol. 2016;173(4):649–65. 25800044 10.1111/bph.13139 PMC4742299

[CR21] Quigley HA, Broman AT. The number of people with glaucoma worldwide in 2010 and 2020. Br J Ophthalmol. 2006;90(3):262–7. 16488940 10.1136/bjo.2005.081224 PMC1856963

[CR23] Reboussin É, Buffault J, Brignole-Baudouin F, Réaux-Le Goazigo A, Riancho L, Olmiere C, et al. Evaluation of neuroprotective and immunomodulatory properties of mesenchymal stem cells in an ex vivo retinal explant model. J Neuroinflammation. 2022;19(1):63. 35236378 10.1186/s12974-022-02418-w PMC8892697

[CR24] Simó R, Villarroel M, Corraliza L, Hernández C, Garcia-Ramírez M. The retinal pigment epithelium: something more than a constituent of the blood-retinal barrier–implications for the pathogenesis of diabetic retinopathy. J Biomed Biotechnol. 2010;2010: 190724. 20182540 10.1155/2010/190724 PMC2825554

[CR25] Singh LP. Thioredoxin interacting protein (TXNIP) and pathogenesis of diabetic retinopathy. J Clin Exp Ophthalmol. 2013. 10.4172/2155-9570.1000287. 24353900 10.4172/2155-9570.1000287 PMC3864179

[CR26] Tezel G. A broad perspective on the molecular regulation of retinal ganglion cell degeneration in glaucoma. Prog Brain Res. 2020;256(1):49–77. 32958215 10.1016/bs.pbr.2020.05.027 PMC11822681

[CR27] Tezel G. Molecular regulation of neuroinflammation in glaucoma: current knowledge and the ongoing search for new treatment targets. Prog Retin Eye Res. 2022;87: 100998. 34348167 10.1016/j.preteyeres.2021.100998 PMC8803988

[CR28] Tsubaki H, Tooyama I, Walker DG. Thioredoxin-interacting protein (TXNIP) with focus on brain and neurodegenerative diseases. Int J Mol Sci. 2020;21(24):9357. 33302545 10.3390/ijms21249357 PMC7764580

[CR29] Wong KY, Roy J, Fung ML, Heng BC, Zhang C, Lim LW. Relationships between mitochondrial dysfunction and neurotransmission failure in Alzheimer’s disease. Aging Dis. 2020;11(5):1291–316. 33014538 10.14336/AD.2019.1125 PMC7505271

[CR30] Xu W, Li T, Gao L, Zheng J, Yan J, Zhang J, et al. Apelin-13/APJ system attenuates early brain injury via suppression of endoplasmic reticulum stress-associated TXNIP/NLRP3 inflammasome activation and oxidative stress in a AMPK-dependent manner after subarachnoid hemorrhage in rats. J Neuroinflammation. 2019;16(1):247. 31791369 10.1186/s12974-019-1620-3 PMC6889224

[CR31] Yuan L, Neufeld AH. Activated microglia in the human glaucomatous optic nerve head. J Neurosci Res. 2001;64(5):523–32. 11391707 10.1002/jnr.1104

[CR32] Zhang X, Zhang Y, Li R, Zhu L, Fu B, Yan T. Salidroside ameliorates Parkinson’s disease by inhibiting NLRP3-dependent pyroptosis. Aging (Albany NY). 2020;12(10):9405–26. 32432571 10.18632/aging.103215 PMC7288953

[CR33] Zhou T, Huang Z, Sun X, Zhu X, Zhou L, Li M, et al. Microglia polarization with M1/M2 phenotype changes in rd1 mouse model of retinal degeneration. Front Neuroanat. 2017;11:77. 28928639 10.3389/fnana.2017.00077 PMC5591873

