# Supplementary figures and images for: Thioredoxin-interacting protein (TXNIP) inhibition promotes retinal ganglion cell survival and facilitates M1-like microglial transformation via the PI3K/Akt pathway in glaucoma
Source: Mol Med. 2024 Dec 30;30:283. doi: 10.1186/s10020-024-01058-5 (PMC11687008; doi:10.1186/s10020-024-01058-5)

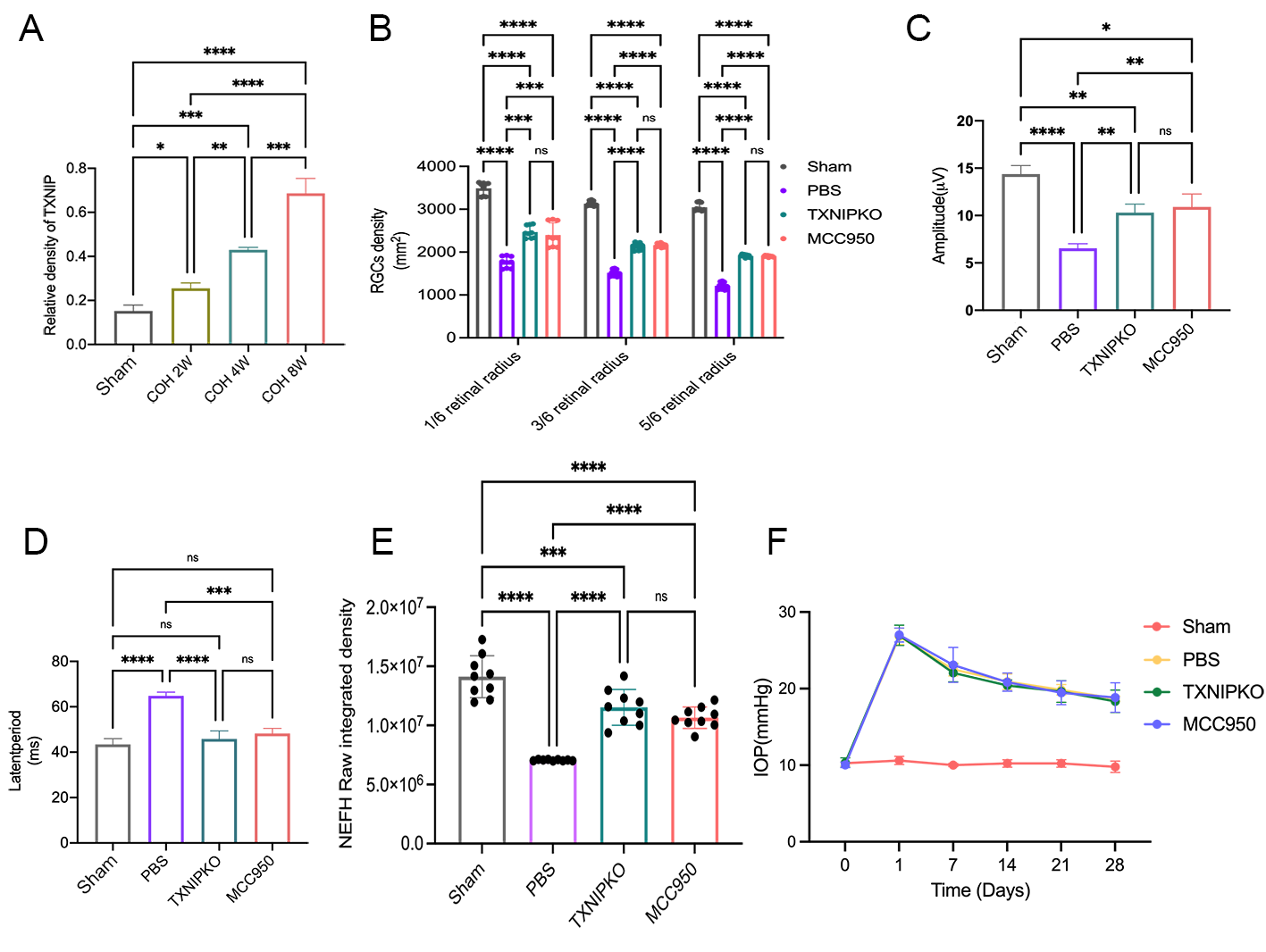

Supplement: Supplementary file 1 — Additional file 1: Figure S1. A Statistical analysis of Western blotting analysis of TXNIP at 0, 2,4, and 8 weeks of COH. B Statistical analysis of immunofluorescent staining of Brn3a in retinal whole mounts at 4 weeks of COH mice in different groups. C, D Statistical analysis of F-VEP test at 4 weeks after COH modeling in different groups. E Statistical analysis of immunofluorescent staining of NEFH in retinal whole mounts at 4 weeks of COH mice in different groups. F Statistical analysis of IOP among 3 groups at different time points. [file 10020_2024_1058_MOESM1_ESM.tif]

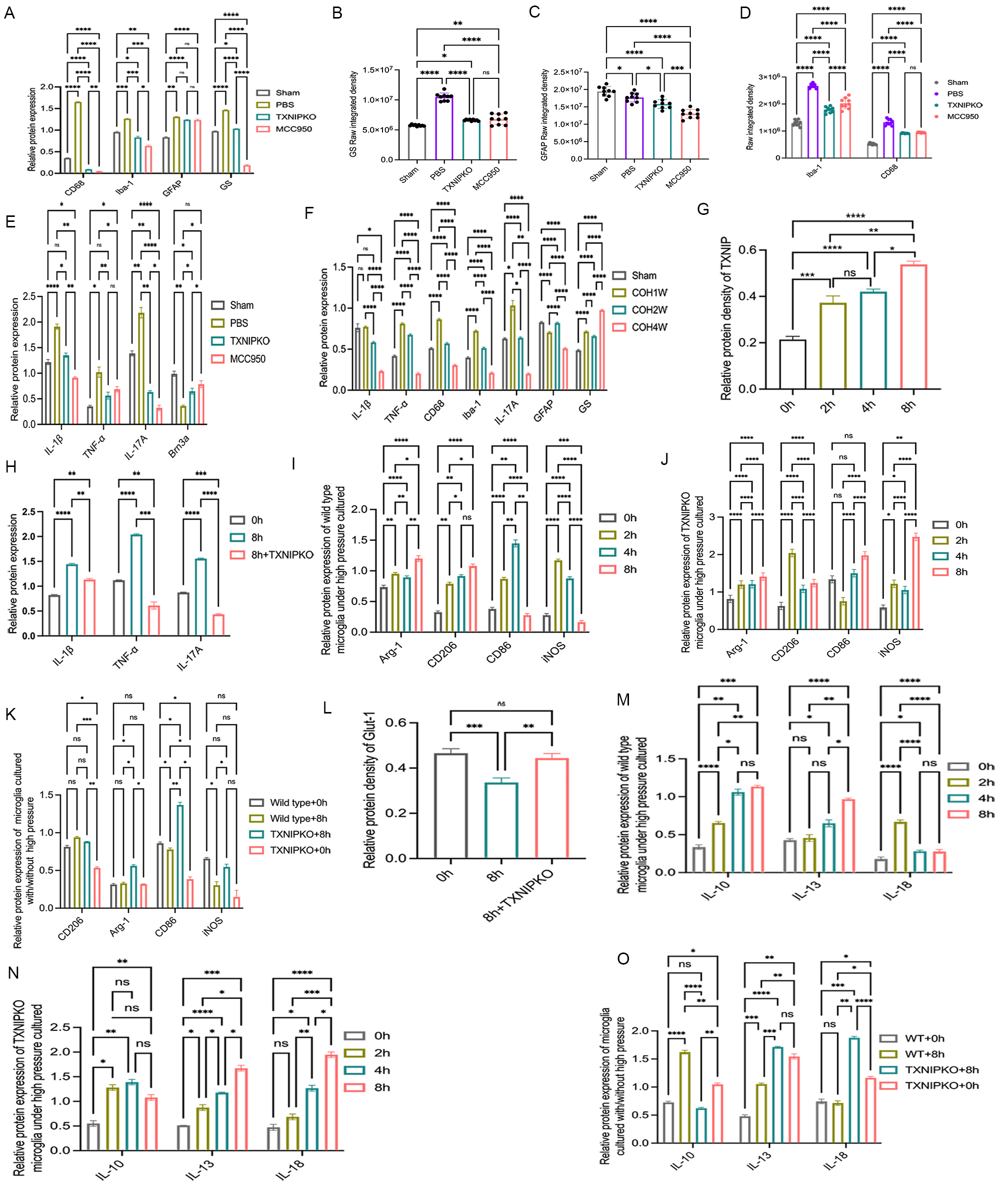

Supplement: Supplementary file 2 — Additional file 2: Figure S2. A Statistical analysis of Western blotting analysis of markers of three glial at 4 weeks of COH in different groups. B, C Statistical analysis of immunofluorescent staining of GFAP and GS in retinal slices at 4 weeks of COH mice in different groups. D Statistical analysis of double immunofluorescent staining of CD68 and Iba-1 in retinal slices of COH mice in different groups. E Statistical analysis of Western blotting analysis of proinflammatory factors and Brn3a at 4 weeks of COH in different groups. F Statistical analysis of Western blotting analysis of proinflammatory factors and cell markers in TXNIP-deficiency COH mice. G Statistical analysis of Western blotting analysis of TXNIP in microglia cultured at 37.5 mmHg. H Statistical analysis of Western blotting analysis of proinflammatory factors in microglia of wild type and TXNIP-deficiency cultured at 37.5 mmHg for 8 h. I Statistical analysis of Western blotting analysis of polarization markers of wild-type microglia cultured at 37.5 mmHg. J Statistical analysis of Western blotting analysis of polarization markers in TXNIP-deficiency microglia cultured at 37.5 mmHg. K Statistical analysis of Western blotting analysis of polarization markers of wild type and TXNIP-deficiency microglia cultured under normal pressure or at 37.5 mmHg for 8 h. L Statistical analysis of Western blotting analysis of the expression of Glut-1 in wild type or TXNIP-deficiency microglia cultured under normal or high pressure for 8 h. M Statistical analysis of Western blotting analysis of IL-10, IL-13, IL-18 of wild-type microglia cultured at 37.5 mmHg. N Statistical analysis of Western blotting analysis of IL-10, IL-13 and IL-18 in TXNIP-deficiency microglia cultured at 37.5 mmHg. O Statistical analysis of Western blotting analysis of IL-10, IL-13 and IL-18 of wild type and TXNIP-deficiency microglia cultured at 37.5 mmHg for 8 h. [file 10020_2024_1058_MOESM2_ESM.tif]

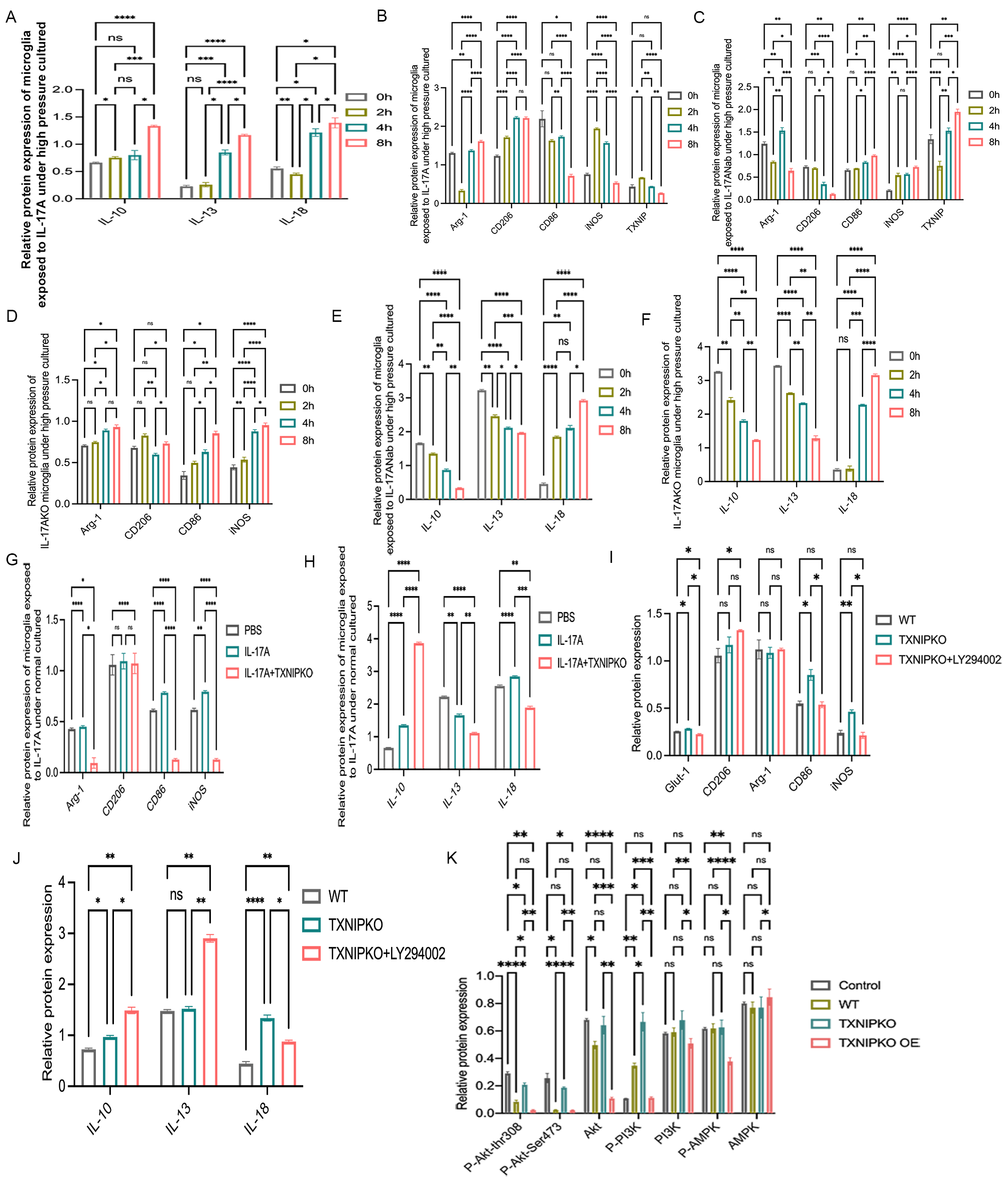

Supplement: Supplementary file 3 — Additional file 3: Figure S3. A Statistical analysis of Western blotting analysis of IL-10, IL-13 and IL-18 of microglia pretreated with rmIL-17A cultured at 37.5 mmHg. B Statistical analysis of Western blotting analysis of M1/M2 cellular markers and TXNIP of microglia pretreated with rmIL-17A cultured at 37.5 mmHg. C Statistical analysis of Western blotting analysis of M1/M2 cell markers of microglia pretreated with IL-17ANab cultured at 37.5 mmHg. D Statistical analysis of Western blotting analysis of M1/M2 cellular markers in IL-17A-deficiency microglia cultured at 37.5 mmHg. E Statistical analysis of Western blotting analysis of IL-10, IL-13 and IL-18 of microglia pretreated with IL-17ANab cultured at 37.5 mmHg. F Statistical analysis of Western blotting analysis of IL-10, IL-13 and IL-18 in IL-17A-deficiency microglia cultured at 37.5 mmHg. G Statistical analysis of Western blotting analysis of M1/M2 cellular markers in wild type or TXNIP-deficiency microglia pretreated with rmIL-17A under normal pressure cultured. H Statistical analysis of Western blotting analysis of IL-10, IL-13 and IL-18 in wild type or TXNIP-deficiency microglia pretreated with rmIL-17A under normal pressure cultured. I Statistical analysis of Western blotting analysis of the expression of Glut-1 and M1/M2 cellular markers in TXNIP-deficiency microglia pretreated with or without LY294002 cultured at 37.5 mmHg. J Statistical analysis of Western blotting analysis of the expression of IL-10, IL-13 and IL-18 in TXNIP-deficiency microglia pretreated with or without LY294002 cultured at 37.5 mmHg. K Statistical analysis of Western blotting analysis of the expression of AMPK and PI3K/Akt signaling pathway proteins in microglia from wildtype or distinctive TXNIP-types cultured at 37.5 mmHg. [file 10020_2024_1058_MOESM3_ESM.tif]
